# S100A10, a novel biomarker in pancreatic ductal adenocarcinoma

**DOI:** 10.1002/1878-0261.12356

**Published:** 2018-09-21

**Authors:** Moamen Bydoun, Andra Sterea, Henry Liptay, Andrea Uzans, Weei‐Yuarn Huang, Gloria J. Rodrigues, Ian C.G. Weaver, Hong Gu, David M. Waisman

**Affiliations:** ^1^ Department of Pathology Dalhousie University Halifax Nova Scotia Canada; ^2^ Department of Physiology and Biophysics Dalhousie University Halifax Nova Scotia Canada; ^3^ Department of Biology Dalhousie University Halifax Nova Scotia Canada; ^4^ Dalhousie Medical School Dalhousie University Halifax Nova Scotia Canada; ^5^ Department of Psychology and Neuroscience Dalhousie University Halifax Nova Scotia Canada; ^6^ Department of Psychiatry Dalhousie University Halifax Nova Scotia Canada; ^7^ Brain Repair Centre Dalhousie University Halifax Nova Scotia Canada; ^8^ Department of Mathematics and Statistics Dalhousie University Halifax Nova Scotia Canada; ^9^ Department of Biochemistry and Molecular Biology Dalhousie University Halifax Nova Scotia Canada

**Keywords:** DNA methylation, KRAS, pancreatic ductal adenocarcinoma, plasminogen, S100A10

## Abstract

Pancreatic cancer is arguably the deadliest cancer type. The efficacy of current therapies is often hindered by the inability to predict patient outcome. As such, the development of tools for early detection and risk prediction is key for improving outcome and quality of life. Here, we introduce the plasminogen receptor *S100A10* as a novel predictive biomarker and a driver of pancreatic tumor growth and invasion. We demonstrated that *S100A10* mRNA and protein are overexpressed in human pancreatic tumors compared to normal ducts and nonductal stroma. *S100A10* mRNA and methylation status were predictive of overall survival and recurrence‐free survival across multiple patient cohorts. *S100A10* expression was driven by promoter methylation and the oncogene *KRAS*. *S100A10* knockdown reduced surface plasminogen activation, invasiveness, and *in vivo* growth of pancreatic cancer cell lines. These findings delineate the clinical and functional contribution of *S100A10* as a biomarker in pancreatic cancer.

**Abbreviations**
CCLE: cancer cell line encyclopedia
CCND1: cyclin D1
DNMT: DNA methyltransferase
ECM: extracellular matrix
FFPE: formalin‐fixed paraffin‐embedded
HM450: human methylation450
HR: hazard ratio
LLC: lewis lung carcinoma
NOD/SCID: nonobese diabetic/severe combined immunodeficiency
OS: overall survival
PanIN: pancreatic intraepithelial
PDAC: pancreatic ductal adenocarcinoma
REV: raw expression value
RFS: recurrence‐free survival
RSEM: RNA‐seq by expression maximization
TCGA: the cancer genome atlas
TMA: tissue microarray
VEGFA: vascular endothelial growth factor A

## 1 Introduction

Pancreatic cancer arises from exocrine or neuroendocrine origin. Pancreatic ductal adenocarcinoma (PDAC) is exocrine in origin and is the predominant form of pancreatic cancers (90%). PDAC is an especially deadly cancer which has a 5‐year survival rate of 4%. Due to early dissemination of pancreatic tumor cells and late manifestation of symptoms, the majority of PDAC patients (92%) are diagnosed with locally advanced or metastatic disease. At that point, surgery is rarely curable and often not recommended to avoid postoperative complications. Although uncommon, early detection may improve 5‐year survival by sixfold (Chari *et al*., 2015). Patients eligible for surgical resection will receive adjuvant chemotherapy with or without radiation which results in a 15–30% chance of surviving to 5 years (Hidalgo, 2010). The gold standard for predicting PDAC patient outcome is tumor, node, metastasis (TNM) staging which performs adequately in late stage (stages III and IV) patients, where their tumors are usually not resectable. However, the prognostic performance of TNM staging is below par in early stage (stages I and II) resectable patients (Helm *et al*., 2009). The consequence of this poor performance is a tendency to undertreat patients who have a high risk of recurrent disease and overtreating patients who are at low risk of recurrence. There is a lack of reliable clinical markers that can identify patients with a high risk of death and/or recurrence. To address such issues, we herein use systematic clinical and functional validation methods to describe a novel biomarker, *S100A10*, and demonstrate its efficacy in predicting PDAC patient outcome.

The initiating genetic event in PDAC is a well‐characterized event. Over 95% of patients have mutations in the oncogene gene *KRAS*. The KRAS protein typically cycles between an inactive guanosine diphosphate (GDP)‐bound conformation and an active GTP‐bound conformation. The latter is capable of dysregulating multiple signaling pathways which drive PDAC oncogenesis and dictate patient outcome. The poor patient prognosis in PDAC is often attributed to high metastatic propensity of cells to colonize metastasis‐prone sites such as the liver, peritoneum, and lungs, ultimately leading to patient mortality. The initial trigger for metastasis occurs at the primary tumor site where a few cancer cells acquire properties that allow them to proteolytically degrade the heavily cross‐linked extracellular matrix (ECM), penetrate the basement membrane, and enter blood vessels (Danø *et al*., 2005; Sevenich and Joyce, 2014). The proteolytic cleavage of ECM proteins is regulated by protease receptors. Our research group has previously demonstrated that S100A10 (p11) is a receptor for the pro‐protease plasminogen and can drive cancer cell invasion by mediating the conversion of plasminogen into the active protease, plasmin (Choi *et al*., 2003; Zhang *et al*., 2004; Phipps *et al*., 2011; Kwon *et al*., 2005; O'Connell *et al*., 2011; Madureira *et al*., 2012; Bydoun and Waisman, 2014). S100A10 binds to annexin A2 in the cytosol forming a heterotetrameric complex which then translocates to the cell surface (Madureira *et al*., 2012). Notably, higher annexin A2 expression has been previously linked to worse outcome in PDAC patients (Zheng *et al*., 2011). We have shown that loss of S100A10 protein results in the inhibition of invasiveness in HT‐1080 fibrosarcoma (Choi *et al*., 2003) and Colo222 colorectal cancer cells (Zhang *et al*., 2004) and decrease in *in vivo* tumor growth of Lewis lung carcinoma (LLC) cells (Phipps *et al*., 2011). The questions of whether S100A10 plays a role in PDAC and whether *S100A10* is a clinically relevant gene are yet to be addressed. Hence, this study has two objectives: first, to utilize cell and mouse models to establish whether S100A10 is involved in the progression of PDAC and, second, to investigate the potential use of *S100A10* as a predictive biomarker. Here, we demonstrate that the protease‐activating function of S100A10 regulates PDAC cell invasion *in vitro* and tumor growth *in vivo*. We also show, for the first time, that *S100A10* mRNA is regulated by DNA methylation both of which are prognostic indicators of overall survival (OS) and recurrence‐free survival (RFS) in PDAC patients.

## 2 Methods

### 2.1 Cell lines and reagents

The Panc‐1 (CRL‐1469, male), Panc10.05 (CRL‐2547, male), and HPAF‐II (CRL‐1997, male) cell lines were purchased from the American Type Culture Collection (ATCC). The AsPC‐1 (female) and Bx‐PC3 (female) cell lines were a generous gift from Dr. David Hoskin (Dalhousie University, Halifax, Nova Scotia, Canada). All cell lines tested negative for mycoplasma. Panc‐1 cells were supplemented with Dulbecco's modified Eagle's media with 10% fetal bovine serum (Hyclone, Logan, UT, USA) and 1% penicillin/streptomycin (Hyclone). AsPC‐1 and BxPC‐3 cells were supplemented with Roswell Park Memorial Institute with 10% fetal bovine serum and 1% pencillin/streptomycin. All cells were maintained at 37 °C with 5% CO_2_. Zarnestra (Tipifarnib; Cat no. S1453, Selleckchem, Houston, TX, USA) and decitabine (Cat no. A3656, Sigma Aldrich, Oakville, ON, Canada) were reconstituted in DMSO. Doxycycline (Cat no. 631311, Clontech, Mountain View, CA, USA) was reconstituted in tissue‐culture grade water. Plasminogen (Cat no. 528180, Sigma Aldrich), S2251 (Via Diapharma, Cat no. 82033239, West Chester, OH, USA), ε‐aminocaproic acid (Cat no. A2504, Sigma Aldrich), and aprotinin (Cat no. 800277, Pentapharm, Dornacherstrasse, Switzerland) were reconstituted in PBS.

### 2.2 Plasmids

The *S100A10* shRNA1 knockdown construct was designed by cloning the following dsRNA oligo (Table S11) into the pSUPER‐retro‐puro vector plasmid (OligoEngine, Seattle, WA, USA). To establish stable *S100A10* knockdown cell lines, Phoenix cells were first transfected with 4 μg of the pSUPER‐retro scramble control and S100A10 shRNA1 plasmids using with lipofectamine 2000 transfection reagent (Cat no. 11668019, Invitrogen, Burlington, ON, Canada). Panc‐1 cells were transduced with the retroviral supernatants and puromycin selection started at 48 h posttransduction. The pBabe‐puro control (#1764) and KRAS^G12D^ (#58902) constructs were obtained the plasmid depository Addgene (Cambridge, MA, USA). The transfected clones were selected in 1 μg·mL^−1^ puromycin.

### 2.3 CDHA patient cohort

Ethics approval was received from the Capital Health Research Ethics Board of Capital District Health Authority (CDHA) on October 09, 2014 (CDHA‐RS/2012‐206). All patients provided written consent for the performed experiments. All methodologies conformed with the standards stated in the Declaration of Helsinki. Eighty‐nine samples were collected from pancreatic adenocarcinoma patients admitted to the Queen Elizabeth Hospital (Halifax, NS) between 2001 and 2009. All patients underwent surgical resection at which point samples were collected prior to adjuvant chemotherapy/radiation. Samples were formalin‐fixed and paraffin‐embedded (FFPE).

### 2.4 Tissue microarray (TMA) construction and immunohistochemistry

Two‐millimeter cores were collected from FFPE blocks and sectioned into 5‐μm sections. Eleven tissue microarrays (TMAs) were constructed of 89 patients with 40 cores/TMA. WYH and AU annotated pancreatic intraepithelials (PanINs) and PDAC regions in each core. Immunohistochemical staining (IHC) was performed with the primary rabbit anti‐human S100A10 antibody (Cat no. 11250‐1‐AP, 1: 800 dilution, Proteintech, Rosemont, IL, USA) using the Ventana automated staining platform (BenchMark ULTRA, Roche, Tuscon, AZ, USA) followed by 3,3′‐diaminobenzidine (DAB) stain.

### 2.5 DAB quantification

**Figure 1:**
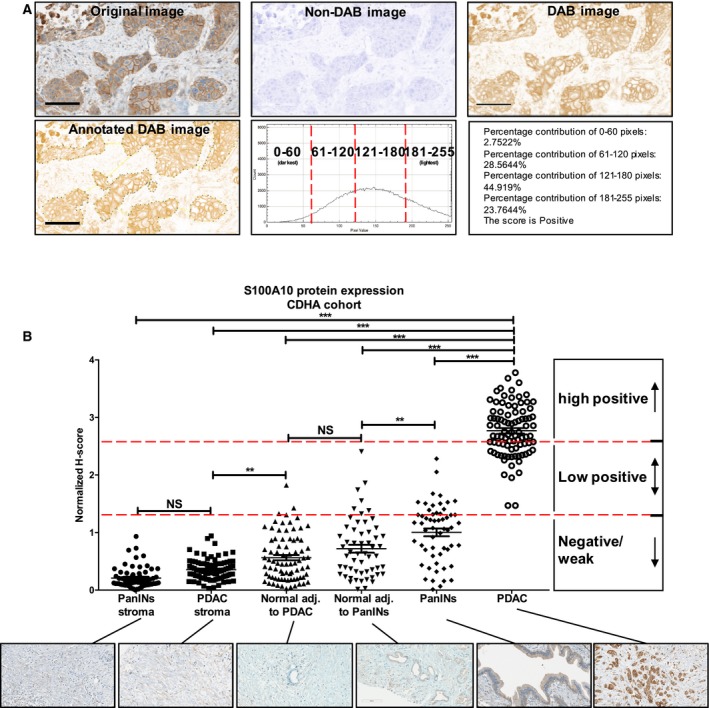
S100A10 protein overexpressed in PDAC compared to PanIN lesions, nonductal stroma, and normal tissue. (A) imagej IHC profiler plugin was used to quantify S100A10 protein expression (see DAB quantification in methods). Briefly, images were color deconvoluted to isolate the brown DAB stain from non‐DAB image. An area of interest (PDAC shown) was manually highlighted and quantified based on pixel intensity and the percentage contribution of each pixel subcategory (0–60, 61–120, 121–180, 181–255; see *H*‐scoring in methods). (B) The graph shows the *H*‐score the S100A10 protein expression quantified by imagej in six different regions: PanINs stroma, PDAC stroma, normal adjacent to PanINs, normal adjacent to PDAC, PanINs, and PDAC lesions. Each *H*‐score was divided by the mean *H*‐score of all measurements to yield a mean‐normalized *H*‐score ± SEM. Significance was determined using one‐way ANOVA of unmatched samples (nonpaired). Scale bars, 100 μm.

Tissue microarray slides were digitized using the Aperio AT2 high volume digital whole slide scanning system (Leica Biosystems, Concord, ON, USA) at 20× magnification. Three representative images of normal, PanIN, and PDAC were captured then subject to color deconvolution in imagej as previously described (Varghese *et al*., 2014). Briefly, color deconvolution separates the hematoxylin (counter stain) from the DAB brown stain. Areas of interest were manually highlighted with the selection tool, and brown stain quantified using the IHC profiler plugin. The profiler generated a pixel intensity histogram from the darkest (intensity value = 0) to the lightest (intensity = 255) shades. Pixel intensity values were divided into four subcategories: 0–60, 61–120, 121–180, and 181–255 (Fig. 1A).

### 2.6 *H*‐scoring

The scoring assignment of selected DAB‐stained areas was accomplished via *H*‐scoring (McCarty *et al*., 1986) using the following formula: *H*‐score = (% of pixels in 0–60 category × 3) + (% of pixels in 61–120 category × 2) + (% of pixels in 121–180 category × 1) + (% of pixels in 181–255 category × 0). *H*‐scores range from 0 to 300. To generate cutoff classifiers, we considered an *H*‐score < 100 to be negative/weak staining, *H*‐score of 100–200 to be low‐positive and *H*‐score of > 200 to be high‐positive values (Table S1). The *H*‐score was then normalized to the average of all intensities.

### 2.7 Kaplan–Meier survival

Survival percentage was calculated nonparametrically based on observed survival times. At the time of last follow‐up, live patients were assigned a zero (0) due to absence of event (i.e., death). Deceased individuals were assigned a one (1) since the event of death occurred. RFS was represented by the duration between a complete response to treatment and the status of disease at time of last follow‐up, that is, disease‐free (0) or progressive disease (1). Log‐rank (Mantel–Cox test) was used to compare relative risk with binary classifiers (median and optimal cutoffs). Multiple comparisons testing was applied to ternary classifier, and an adjusted *P*‐value was calculated based on a Bonferroni‐corrected threshold. The adjusted *P*‐value represents the number of comparisons made; *P*‐value_adj_ = *P*‐value_raw_/*k*, where raw *P*‐value = 0.05 and *k* = 3.

### 2.8 Univariate and multivariate analysis

Univariate and multivariate regression models were fitted to the overall (OS) and RFS of the the cancer genome atlas (TCGA) PDAC patient cohort. The variables/predictors are *S100A10* mRNA [RNA‐seq V2 RNA‐seq by expression maximization (RSEM)], gender, race, age, grade, tumor dimension, stage, metastasis, smoking, and alcohol consumption. A natural logarithm (ln) was applied to the *S100A10* mRNA raw expression values (REVs). The fitted single‐variable model included all variables listed. The fitted multivariate model included all variables except smoking history and alcohol consumption due to high number of missing values on these two variables. A semi‐parametric proportional hazard regression model was fitted to identify variables that are predictors of survival time. The model assumes *h*(*t*|*Z*) = *h*0(*t*) exp(β′ *Z*), where *h*(*t*|*Z*) is the hazard rate at time t for an individual with risk vector *Z*,* h*0(*t*) is an arbitrary baseline hazard rate, β is a vector of coefficients, and *Z* is a vector of covariants or variables. We fitted the semi‐parametric proportional hazards regression model for each single variable. The univariate and multivariate analyses’ results are summarized in Tables S4 through S8.

### 2.9 *In vivo* intraperitoneal mouse model

5 × 10^6^ Panc‐1 cells (scramble control or *S100A10*‐shRNA 1) were suspended in PBS and intraperitoneally injected into the lower right abdominal area of alert nonobese diabetic‐severe combined immunodeficiency (NOD‐SCID) mice. After 70 days postinjection, tumors were collected, weighed, fixed with 10% formalin, and embedded in paraffin for histological examination. The animal experiment studies were approved by Dalhousie Animal Ethics (Protocol Number 15‐143) and housed at the Carlton Animal Care Facility. Animal weight was monitored weekly to assess well‐being. The 70‐day endpoint was chosen to maintain humane endpoint with minimal weight loss and no effect on mobility.

### 2.10 Gene expression data normalization

RNA seq V2 RSEM expression values for the TCGA tumors (Fig. S1A) as well as cancer cell line encyclopedia (CCLE) *Z*‐scores (Fig. S1B) were downloaded from CBioPortal. RNA seq V2 REVs were normalized by dividing by the mean expression value (Li and Dewey, 2011). *Z*‐scores were compared using the *z*‐ratio equation as previously described (Cheadle *et al*., 2003). *z*‐ratio = *z*‐score_avg_ (cell type) − *z*‐score_avg_ (CML)/SD of *z*‐score differences. *z*‐score_avg_ (cell type) is the average of the *z*‐scores of all the cell lines within a particular tumor type (CML: chronic myelogenous leukemia). *z*‐score_avg_ (CML) is the average of the *z*‐scores of CML cell lines which had the lowest average *z*‐score and was used as a control. SD of *z*‐score differences is the standard deviation (SD) of the [*z*‐ratio = *z*‐score_avg_ (cell type) − *z*‐score_avg_ (CML)] values of each tumor type. A *z*‐ratio of 1.96 or higher is considered equivalent to a *P*‐value = < 0.05. For normal/tumor data normalization, expression values were retrieved from the Gene expression Omnibus (GEO) as per corresponding accession numbers http://www.ncbi.nlm.nih.gov/protein/GSE16515 (Pei *et al*., 2009), http://www.ncbi.nlm.nih.gov/protein/GSE22780 (Balasenthil *et al*., 2011), http://www.ncbi.nlm.nih.gov/protein/GSE3654 (Donahue *et al*., 2012), http://www.ncbi.nlm.nih.gov/protein/GSE1542 (Ishikawa *et al*., 2005), http://www.ncbi.nlm.nih.gov/protein/GSE15471 (Badea *et al*., 2008), and http://www.ncbi.nlm.nih.gov/protein/GSE28735 (Zhang *et al*., 2012) log‐transformed and median‐centered per array (Fig. S2). Expression values from Segara *et al*. (2005) and Logsdon *et al*. (2003) gene arrays were extracted from Oncomine (Rhodes *et al*., 2007) as median‐centered intensities.

### 2.11 Bisulfite conversion and pyrosequencing

As previously described (Delaney *et al*., 2015), DNA methylation was analyzed by sodium bisulfite pyrosequencing on a pyromark Q24 Advanced pyrosequencer using the DNA EpiTect Fast DNA Bisulfite Kit and pyromark PCR Kit (Cat no. 978703, Qiagen, Germantown, MD, USA) as per manufacturer's instructions beginning with 500 ng template DNA. A custom assay covering the region immediately upstream of the *S100A10* gene transcription start site (TSS) was designed using pyromark Assay Design software (v2.0; Qiagen) and validated to amplify a single PCR product (417 nt). Primers are listed in Table S11. PCR conditions for both assays: 95 °C, 15 min; (94 °C, 30 s; 56 °C, 30 s; 72 °C, 30 s) × 50 cycles; and 72 °C, 10 min.

### 2.12 Quantitative RT‐PCR

RNA was extracted using TRIzol as per standard procedure (Qiagen). Two microgram of RNA was used for the synthesis of cDNA using Superscript II (Invitrogen). Gene expression was amplified using gene‐specific primers on the CFX96™ platform. The primers were designed with high specificity and verified for optimal amplification (Table S11). Relative mRNA expression was calculated using the Livak and Schmittgen's 2^−∆∆CT^ method with GAPDH or β‐actin as reference genes (Livak and Schmittgen, 2001).

### 2.13 Western blotting

Cells were lysed in lysis buffer (1% NP‐40, 150 mm NaCl, 20 mm Tris, pH 7.0, 1 mm EDTA, and 1 mm EGTA) containing 2× Halt protease and phosphatase inhibitors (ThermoScientific, Waltham, MA, USA). Samples were subject to SDS/PAGE then transferred onto a nitrocellulose membrane. Membranes were incubated with primary antibodies overnight at 4 °C or 1 h at room temperature. Li‐COR secondary antibodies used to visualize bands using a LI‐COR Odyssey imaging scanner. Relative band intensities per lane were determined for each protein and normalized to intensities of β‐actin bands.

### 2.14 MTS assay

1 × 10^4^ cells were seeded in a 96‐well plate. Twenty microliter of Promega's CellTiter 96 one solution reagent (Cat no. G3582, Promega, Madison, WI, USA) was added to 100 μL of the culture medium and incubated for 4 h at 37 °C after which the amount of soluble formazan was measured by recording the absorbance at 490 nm using the SpectraM3 plate reader.

### 2.15 Plasminogen activation assay

Panc‐1 and iKRAS cells were seeded overnight into 96‐well plates at 5 × 10^3^ and 1 × 10^5^ cellsper well respectively. Cells were then washed with PBS (Hyclone), incubated with 0.5 μm plasminogen for 10 min, and then incubated with 0.5 mm S2251. Plasminogen activation was measured based on the absorbance of cleaved S2251 at 405 nm every 4 min for 4 h using the Spectra M3 plate reader (Molecular Devices).

### 2.16 Invasion assay

5 × 10^4^ Scramble control and *S100A10*‐shRNA1 Panc‐1 cells were seeded in serum‐free media into the upper chamber of a trans‐well Boyden chamber with 8 μm pores (BD Biosciences, San Jose, CA, USA). The bottom chamber contained 10% FBS as a chemoattractant. 0.5 μm plasminogen was added to the top chambers 5 h after seeding. After 72 h, transversed cells were stained with hematoxylin and eosin and counted (five fields of view per membrane at 20× magnification).

### 2.17 Ras activation assay

Protein lysates from vehicle‐ and Zarnestra‐treated Panc‐1 and BxPC‐3 cells were incubated with a Raf‐1 pulldown reagent linked to agarose beads as per manufacturer's instructions (Cat no. 16117, EMDMillipore, Etobicoke, ON, Canada). Lysates were then separated on an SDS/PAGE and immunoblotted using a RAS antibody (Cat no. 05‐516 Clone RAS10, EMDMillipore).

### 2.18 Statistical analysis

All experiments were performed in triplicate in three independent experiments. All statistical analyses were performed using graphpad prism 5 software (La Jolla, CA, USA). Unless indicated in the figure legends, statistical significance was determined using the unpaired Student *t*‐test or one‐way ANOVA. A significance threshold of *P*‐value < 0.05 was used with the exception of multiple comparisons tests (*P*‐value < 0.017). Significance was represented using asterisks; *P‐value < 0.05, **P‐value < 0.01, ***P‐value < 0.001, ****P‐value < 0.0001.

## 3 Results

### 3.1 *S100A10* mRNA is highly expressed in pancreatic tumors and cell lines

To assess the relative expression levels of the *S100A10* gene in cancer, we examined *S100A10* mRNA levels (RNA seq V2 RSEM) across all 33 cancer types in the genomics data commons portal of the National Cancer Institute (Grossman *et al*., 2016). *S100A10* mRNA expression in PDAC (*n* = 179) was the third highest (mean = 1.959, CI: 1.789–2.129) after mesothelioma (*n* = 87; mean = 3.895, CI: 3.501–4.290) and head and neck squamous cell carcinoma (*n* = 801; mean = 2.030, C.I 1.951–2.109; Fig. S1A). We also examined *S100A10* mRNA levels (microarray *z*‐scores) across all 930 human cancer cell lines listed in the CCLE from the Broad Institute (http://www.ncbi.nlm.nih.gov/protein/GSE36133) (Barretina *et al*., 2012). *S100A10* was highly expressed in many cancer cell lines including upper respiratory tract (*n* = 30; mean = 0.6671 C.I 0.6314–0.7029), pancreatic (*n* = 44; mean = 0.6657, CI: 0.5948–0.7366), and esophageal (*n* = 25; mean = 0.6542, CI: 0.5838–0.7245) cancer cell lines (Fig. S1B). These results established that *S100A10* mRNA is highly expressed in many cancer types including pancreatic tumors and cell lines suggesting a possible role of *S100A10* in PDAC.

### 3.2 S100A10 is highly expressed in pancreatic tumors compared to adjacent nonductal stroma and normal ducts

After establishing that *S100A10* mRNA was highly expressed in pancreatic tumors and cell lines, we focused on studying its relevance in this cancer. We compared *S100A10* mRNA expression in normal and tumor samples from previously published DNA microarray and RNA seq expression datasets. A consistent upregulation of *S100A10* mRNA was observed in pancreatic tumors compared to normal tissues of unmatched (Fig. S2A–F) and matched (Fig. S2G–I) patients.

To gain further insight into *S100A10* expression in pancreatic tumors beyond mRNA levels, we examined protein expression in 89 archived human pancreatic tumors using immunohistochemistry (IHC). The additional benefit of IHC is the ability to discern the anatomical structure and cell types that are the source of the S100A10 protein signal. Consistent with our mRNA analysis, S100A10 protein expression was also upregulated in cancerous regions compared to nearby normal ducts. There were regions of intense S100A10 staining which correlated with neoplastic cells and regions of weak staining which corresponded to apparently normal cells within a single duct (Fig. S3). We then constructed TMAs of the entire PDAC cohort. Control, PanIN, and PDAC sections were annotated and stained with an anti‐S100A10 antibody which was quantified by imagej (Fig. 1A; Table S1; see Methods). Weak/negative staining was observed in 0% (0/88) of PDAC, 66.67% (38/57) of PanINs, 94.94% (75/79) of normal ducts adjacent to PDAC, 87.50% (49/56) of normal duct adjacent to PanINs, 100% (88/88) of PDAC nonductal stroma, and 100% (63/63) of nonductal PanINs stroma. Low‐positive staining was observed in 34.09% (30/88) of PDAC, 33.33% (19/57) of PanINs, 5.06% (4/79) of normal ducts adjacent to PDAC, and 12.50% (7/56) of normal duct adjacent to PanINs. Importantly, we observed that high‐positive staining was exclusive to PDAC at 65.91% (58/88; Fig. 1B). Therefore, S100A10 protein is overexpressed in carcinoma (PDAC) regions compared to PanINs, normal ducts, and nonductal stroma. Collectively, both mRNA and protein levels of S100A10 revealed a similar trend of upregulation in tumor tissue compared to normal tissue.

### 3.3 *S100A10* mRNA expression and copy number are predictive of overall and recurrence‐free survival in PDAC patients

Having established S100A10 upregulation in PDAC, we examined the potential clinical significance of S100A10 in the prognosis of the TCGA provisional PDAC patient cohort (*n* = 178). Kaplan–Meier survival analysis was performed on patients using three cutoff classifiers (median cutoff, optimal cutoff, and ternary cutoff; Fig. S4A–C). A median cutoff (REV > or < median) revealed that *S100A10* mRNA expression is predictive of both OS [hazard ratio (HR) = 2.16, *P*‐value = 0.0003, *n* = 178] and RFS (HR = 2.42, *P*‐value < 0.0001, *n* = 139). High‐*S100A10* mRNA levels predicted poorer long‐term survival, and patients were more likely to recur over the 90‐month follow‐up period (Fig. S4B,C). In addition, 1‐, 3‐, and 5‐year survival in low‐*S100A10* patients (e.g., 1 year OS: 69.66%, 1‐year RFS: 58.57%) was significantly higher than that in high‐*S100A10* patients (e.g., 1‐year OS: 59.55%, 1‐year RFS: 49.28%; Table S2).

**Figure 2:**
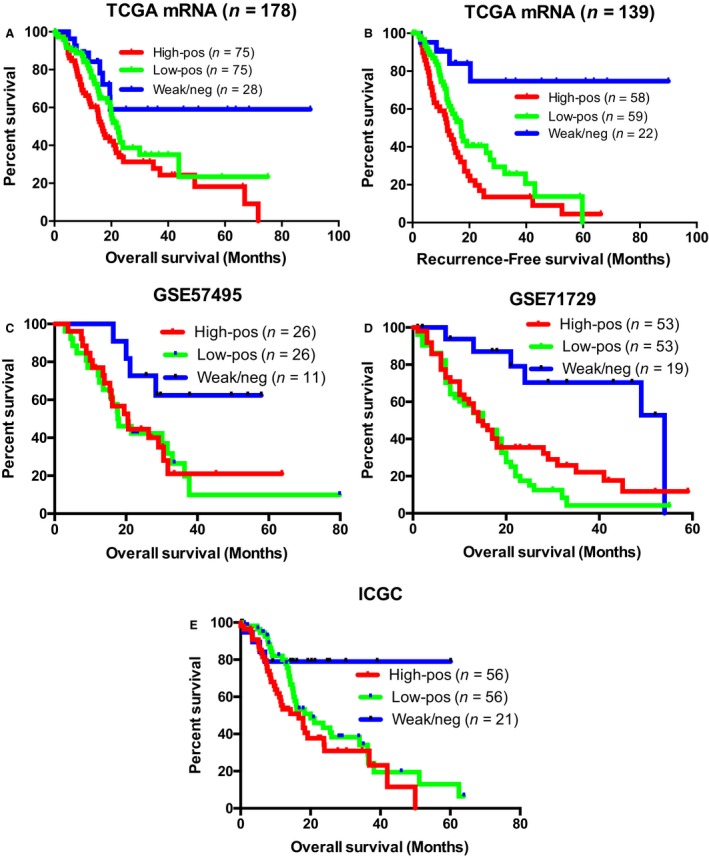
S100A10 mRNA expression is predictive of overall and RFS in four PDAC patient cohorts. Kaplan–Meier (KM) plots of OS (A,C–E) and RFS (*n* = 139; B) of PDAC patients based on their S100A10 mRNA expression. Patients in (A,B) are from the TCGA provisional cohort. Patients in (C,D,E) are derived from Chen *et al*. (2015, http://www.ncbi.nlm.nih.gov/protein/GSE57495), Moffitt *et al*. (2015, http://www.ncbi.nlm.nih.gov/protein/GSE71729), and ICGC. The ternary cutoff was applied to classify the high‐positive, low‐positive, and weak/negative subgroups. *P*‐values were adjusted to the Bonferroni‐corrected threshold. Adjusted *P*‐value is *P*‐value/*K* = 0.017 where *K* = 3 and represents the number of comparisons made (Table S3).

Although a median cutoff resulted in a strong correlation between OS and RFS and *S100A10* mRNA expression, we attempted to utilize a more optimal cutoff that would allow a strict binary classification of high and low expressors. The cutoff finder tool previously described by Budczies *et al*. (2012) identified a new binary classifier of a high‐risk group (93.82%) with high expression of *S100A10* mRNA (REV > 3790.9211) and a low‐risk group (6.18%) with considerably low expression of *S100A10* mRNA (REV ≤ 3790.9211). The low‐risk group had a very favorable long‐term OS and RFS (Fig. S4B). To further test the prognostic performance of S100A10 and bypass the conservative and biased approach of optimal cutoffs, we developed a ternary classifier based on the frequency distribution of REVs in the TCGA cohort (Fig. S4C). The ternary classification identified three subgroups of patients: a weak/negative group with a favorable OS and RFS outcome and two largely indifferent groups (low‐positive and high‐positive) with less favorable outcome (Fig. 2A,B; Table S3). We then applied the same ternary classifier to three additional independent PDAC studies: Chen *et al*. (2015) (http://www.ncbi.nlm.nih.gov/protein/GSE57495, *n* = 63), Moffitt *et al*. (2015) (http://www.ncbi.nlm.nih.gov/protein/GSE71729, *n* = 125), and International Cancer Genome Consortium (ICGC, *n* = 133; Zhang *et al*., 2011). Kaplan–Meier survival curves revealed a similar trend of survivability to that observed in the TCGA PDAC cohort. An equivalent low‐risk group with favorable OS emerged in Chen *et al*. (Fig. 2C, *P*‐value = 0.0402), Moffitt *et al*. (Fig. 2D, *P*‐value = 0.0026), and ICGC (Fig. 2E, *P*‐value = 0.0073) cohorts when compared to the high‐positive group (Table S3). Collectively, these survival analyses showed that low expression of *S100A10* mRNA can serve as a strong predictor of favorable short‐ and long‐term survival in PDAC patients.

*S100A10* mRNA expression also significantly correlated with its copy number score (Fig. S5A) and status (Fig. S5B) in TCGA PDAC patients. As the Kaplan–Meier analysis of *S100A10* mRNA expression correlated with OS and RFS of PDAC patients within the TCGA cohort, we examined whether *S100A10* gene copy number showed similar correlations. Higher *S100A10* copy number score correlated with poorer OS (HR = 1.816, *P*‐value = 0.0357, *n* = 176; Fig. S5C) and RFS (HR = 1.691, *P*‐value = 0.0190, *n* = 139; Fig. S5D). Short‐term OS and RFS also correlated with *S100A10* copy number score (Table S2). In attempt to complement the copy number score‐based stratification, patients were also stratified based on *S100A10* copy number status (i.e., deletion, diploid, gain, or amplification). Patients with *S100A10* amplifications had a noticeably shorter OS and RFS compared to patients with *S100A10* deletions (Fig. S5E,F). The usage of mRNA levels as a predictive marker is supported by the observation that *S100A10* copy number also possessed similar predictive potential within the same cohort.

### 3.4 *S100A10* mRNA and lymph node positivity are linked predictors of overall and recurrence‐free survival

To understand the relationship between *S100A10* mRNA and other clinical covariates, we applied univariate and multivariate regression models. Based on the Wald test, single variable analysis indicated that five variables were predictive of OS: *S100A10* mRNA (HR = 1.79, CI: 1.30–2.46, *P*‐value = 0.00038), age (HR = 1.03, CI: 1.01–1.05, *P*‐value = 0.008), Grade II (HR = 2.00, CI: 1.07–5.08, *P*‐value = 0.041), Grade III (HR = 2.55, CI: 1.26–5.14, *P*‐value 0.009), lymph node positivity (HR = 2.09, CI: 1.24–3.51, *P*‐value = 0.005), and Stage II (HR = 2.33, CI: 1.07–5.08, *P*‐value = 0.03). Although age as a single variable was a significant predictor of OS, the HR was marginal (Table S4). The likelihood ratio test for all five variables revealed that only *S100A10* mRNA, age, and lymph node positivity were significant. The multivariate regression fitting further confirmed the prognostic significance of *S100A10* mRNA (HR = 1.58, CI: 1.07–2.35), lymph node positivity (HR = 2.18, CI: 1.09–4.35), and age (HR = 1.02, CI: 1.001–1.044; Table S5). An ANOVA test of these three variables validated their predictive power (*P*‐values: 0.007, 0.003, and 0.034 respectively). A final model using these three variables was then derived which shows that for every unit increase in *S100A10* mRNA REV (log of the *S100A10* mRNA REV increases by one is equivalent to the *S100A10* mRNA REV increases by 2.718 times of the original level), the likelihood of dying is 1.54 higher (CI: 1.07–2.21, *P*‐value = 0.02). Similarly, being lymph node‐positive increases the risk of death by 1.93 times (CI: 1.15–3.24, *P*‐value = 0.01). The effect of age on this model is minor although statistically significant. The risk of death is 2.97 times higher in lymph node‐positive patient with one‐unit increase in *S100A10* mRNA (i.e., REV = Y) compared to a lymph node‐negative patient with lower *S100A10* mRNA (REV = X; Table S8).

Univariate and multivariate regression models of RFS functions were also generated. The single variable analysis using the Wald test showed that *S100A10* mRNA (HR = 2.12, CI: 1.52–2.94, *P*‐value = 7.89e‐06), Grade II (HR = 2.14, CI: 1.08–4.23, *P*‐value 0.029), Grade III (HR = 3.29, CI: 1.61–6.71, *P*‐value = 0.001), and lymph node positivity (HR = 1.79, CI: 1.10–2.94, *P*‐value = 0.018) were predictive of RFS (Table S6). The likelihood ratio test rendered *S100A10* mRNA, grade, and lymph node positivity as the only significant variables. Subsequent multivariate analysis revealed that only *S100A10* mRNA (HR = 1.71, CI: 1.12–2.61) and lymph node positivity (HR = 1.96, CI: 1.00–3.84) were the significant predictors of RFS (Table S7). ANOVA tests confirmed this result with *P*‐values 0.0003 and 0.02, respectively, for *S100A10* mRNA and lymph node positivity. Thus, a final two‐variable model was derived which predicts the likelihood of recurrence as 1.89 times higher for every unit increase in *S100A10* mRNA. The recurrence rate also increases by 1.54 times in lymph node‐positive patients. Consequently, a lymph node‐positive patient with one‐unit increase in *S100A10* mRNA is 2.915 times more likely to recur than a lymph node‐negative patient with lower *S100A10* mRNA (Table S8). These results established that *S100A10* mRNA and lymph node status are linked covariates and are strong predictors of OS and RFS in PDAC patients.

### 3.5 S100A10 methylation status is predictive of overall and recurrence‐free survival in PDAC patients

**Figure 3:**
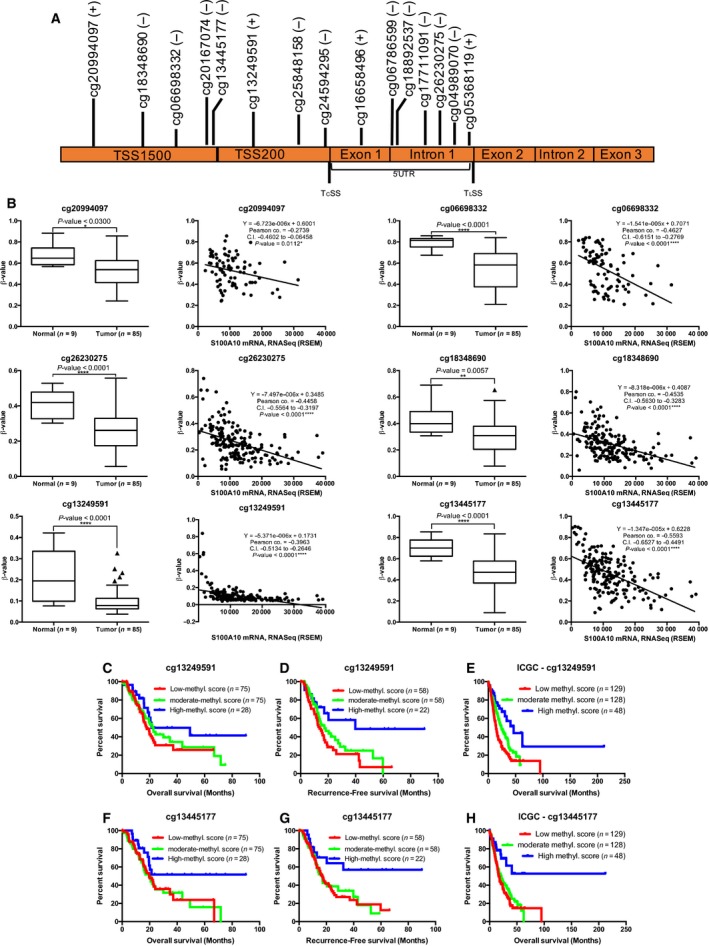
Differentially methylated CpG sites negatively correlate with S100A10 mRNA expression and serve as predictors of survival. (A) Schematic illustration of the human S100A10 gene based on UCSC Ref‐Seq. The genomic distance is approximate but is not drawn to scale. T_c_SS, transcription start site; T_L_SS, translation start site; TSS1500, region between 200 bp and 1500 bp upstream of T_c_SS; TSS200, region 200 bp upstream of T_c_SS; 5′UTR, 5′ untranslated region. The *S100A10* gene is encoded on the negative strand (−), four probes mapped to the opposite positive (+) strand. Five probes were mapped to TSS1500, three to TSS200, and seven probes to the 5′UTR. (B) For normal vs. tumor comparisons, the raw data were extracted from MethHC (http://methhc.mbc.nctu.edu.tw/php/index.php), described by Huang *et al*. (2015). The β‐values of each probe were assessed in 85 PDAC tumors and nine normal tissues (first and third columns). For mRNA vs. methylation correlations, raw β‐values of individual probes were extracted from Maplab Wanderer (http://maplab.imppc.org/wanderer/) (Díez‐Villanueva *et al*., 2015) and plotted against RNA seq V2 (RSEM) expression values of S100A10 in matched patients. Pearson's correlation was used to generate correlation graphs of β‐values and S100A10 mRNA expression (second and fourth columns). β‐Values for the probe cg06786599 were absent for normal samples, and no significant correlation (*P*‐value = 0.1023) between S100A10 tumor mRNA and cg06786599 β‐values was found. Cg06786599 was then excluded from further analysis. Significance was determined using unpaired Tukey test. Data are represented as mean ± SD. Kaplan–Meier (KM) plots of OS (*n* = 178; C,F) and RFS (*n* = 139; D,G) based on β‐values of the cg13249591 and cg13445177 probes. Overall survival was also assessed in the ICGC cohort was assessed based on the β‐values of both probes (E,H). *P*‐values were adjusted to the Bonferroni‐corrected threshold. Adjusted *P*‐value is *P*‐value/*K* = 0.017 where *K* = 3 and represents the number of comparisons made (Table S11).

The availability of human methylation450 (HM450) methylation data of the TCGA cohort enabled us to address the methylation status of the *S100A10* gene and, importantly, its correlation with *S100A10* mRNA. Fifteen probes mapped the *S100A10* gene and promoter regions as illustrated in Fig. 3A. We also identified all the CpG sites corresponding to each probe (Table S9). As mRNA and protein levels were significantly higher in PDAC tumors compared to normal tissue, we examined the TCGA HM450 β‐values for both normal (*n* = 9) and tumor (*n* = 85) tissues (Huang *et al*., 2015). Six probes met the criteria of 1) being differentially hypomethylated in tumor tissue compared to normal tissue and 2) negatively correlated with *S100A10* mRNA expression (Fig. 3B). The remaining probes were not hypomethylated in tumors and/or did not negatively correlate with mRNA expression (Fig. S6). The third criterion was to discern which of the six probes was predictive of survival in the PDAC cohort. Kaplan–Meier survival analysis (Figs S7 and S8) using the ternary classifier showed that high β‐values of the probes cg13249591 and cg13445177 predicted a low‐risk group of patients that had favorable OS (Fig. 3C,F) and RFS (Fig. 3D,G) when compared to the groups with moderate and low methylation scores which were had similar outcomes (Table S10). Noteworthy, under the optimal cutoff conditions, there was an 81.82% (9/11) patient concordance in the low‐risk groups and 98.8% (165/167) in the high‐risk groups between mRNA and cg13445177 methylation assessments of OS. Meanwhile, RFS assessment revealed 90% (9/10) and 99.22% (128/129) concordances in the low‐risk and high‐risk groups, respectively (Fig. S9B,D). We then assessed both probes in the ICGC methylation dataset using the same ternary classifier which showed a similar OS pattern (Fig. 3E,H). To ensure that the high β‐values in the patient subgroup with high methylation scores were not due to global increase in DNA methylation by the *de novo* methyl transferases (Jin and Robertson, 2013), we compared the mRNA expression of these DNA methyltransferases (DNMTs) with β‐values of the two probes. No noticeable correlation was observed between the two probes and mRNA expression of *DNMT1*,* DNMT3A,* or *DNMT3B* (Fig. S10A,B).

### 3.6 S100A10 expression is regulated by methylation at specific CpG sites in the promoter region

**Figure 4:**
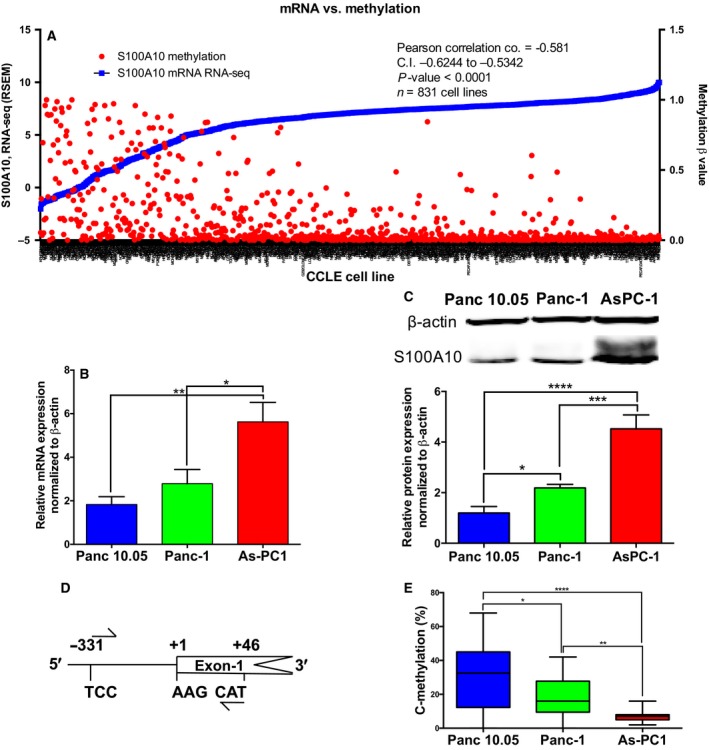
S100A10 mRNA and protein expression negatively correlated with promoter methylation in PDAC cell lines. (A) The relationship between S100A10 methylation and mRNA expression in 831 CCLE cell lines. mRNA expression (RNA seq V2 RSEM) and methylation (RRBS β‐values) were extracted from the broad institute CCLE portal (https://portals.broadinstitute.org/ccle). S100A10 mRNA (RT‐qPCR; B) and protein expression (C) in three PDAC representative cell lines: Panc 10.05, Panc‐1, and AsPC‐1. (D) S100A10 promoter construct for bisulfite and pyrosequencing covering 24 CpG dinucleotides. (E) Global methylation of the 24 CpGs in the S100A10 promoter. The graph represents the averages of percentages of all 24 sites in each cell line. Significance was determined using one‐way ANOVA. Data are represented as mean ± SD.

**Figure 5:**
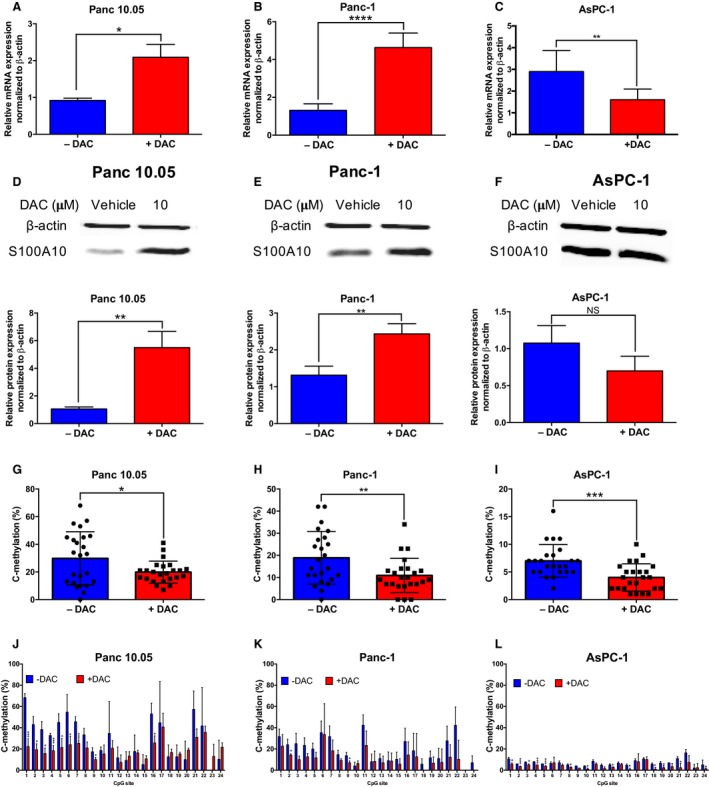
S100A10 mRNA expression is regulated by differential CpG site methylation. S100A10 mRNA (A,B,C) and protein (D,E,F) changes in Panc 10.05 (A,D), Panc‐1 (B,E), and AsPC‐1 (C,F) in response to 10 μm decitabine (DAC) for 72 h. Global and CpG‐specific methylation of the 24 CpGs in the S100A10 promoter in Panc 10.05 (G,J), Panc‐1 (H,K), and AsPC‐1 (I,L). Graphs G–I represent the averages of percentages of all 24 sites in each cell line. Graphs J–L represent the percentage methylated of cytosines of a specific CpG site within each sample. Significance was determined using unpaired *t*‐tests. Data are represented as mean ± SD.

To validate that *S100A10* is regulated by DNA methylation *in* *cellulo*, we first compared *S100A10* mRNA expression in the CCLE cell lines. A negative correlation between *S100A10* mRNA (RNA seq V2 RSEM) and DNA methylation was observed across all cell lines (Pearson's correlation coefficient = −0.581; Fig. 4A) including pancreatic cell lines (Fig. S11A). We then compared *S100A10* mRNA and protein levels and promoter methylation in three cell lines that are representative of expression/methylation spectrum (Panc 10.05, Panc‐1 and AsPC‐1). Panc10.05 cells had the lowest *S100A10* mRNA (Fig. 4B) and protein expression (Fig. 4C) followed by Panc‐1 and AsPC‐1 cells. To examine whether the *S100A10* promoter region was differentially methylated in the three‐cell line panel, we performed bisulfite conversion followed by pyrosequencing of a 377‐nucleotide promoter region containing 24 CpG sites (Fig. 4D; Fig. S11B). Consistent with the mRNA levels, global DNA methylation of that region was the highest in Panc 10.05 cells followed by Panc‐1 and AsPC‐1 cells (Fig. 4E). Notably, AsPC‐1 cells had considerably higher mRNA and protein levels and significantly low DNA methylation. To address effect of DNA demethylation on S100A10 expression, all three cell lines were treated with the DNA demethylating agent decitabine. *S100A10* mRNA and protein levels were dramatically upregulated in Panc 10.05 (Fig. 5A,D) and to a lesser extent in Panc‐1 cells (Fig. 5B,E). In contrast, no increase was observed in the AsPC‐1 cell line (Fig. 5C,F). Despite the differential response in *S100A10* mRNA, the overall methylation of the promoter region was further decreased in all three cell lines in response to decitabine (Fig. 5G,H,I). Such decrease was also seen across the individual CpG sites examined (Fig. 5J,K,L). Notably, the cg13445177 and cg13249591 probes mapped CpG sites 6 and 7 and sites 9 and 10, respectively. Only CpG‐9 was differentially demethylated across all three cell lines, indicating that this site (in addition to others) was likely responsible in sustaining low *S100A10* mRNA in PDAC patients. Collectively, these results indicated that *S100A10* expression is regulated through hypomethylation at specific CpG sites.

### 3.7 S100A10 acts as a plasminogen receptor at the surface of pancreatic cancer cells and contributes to cancer cell invasion

**Figure 6:**
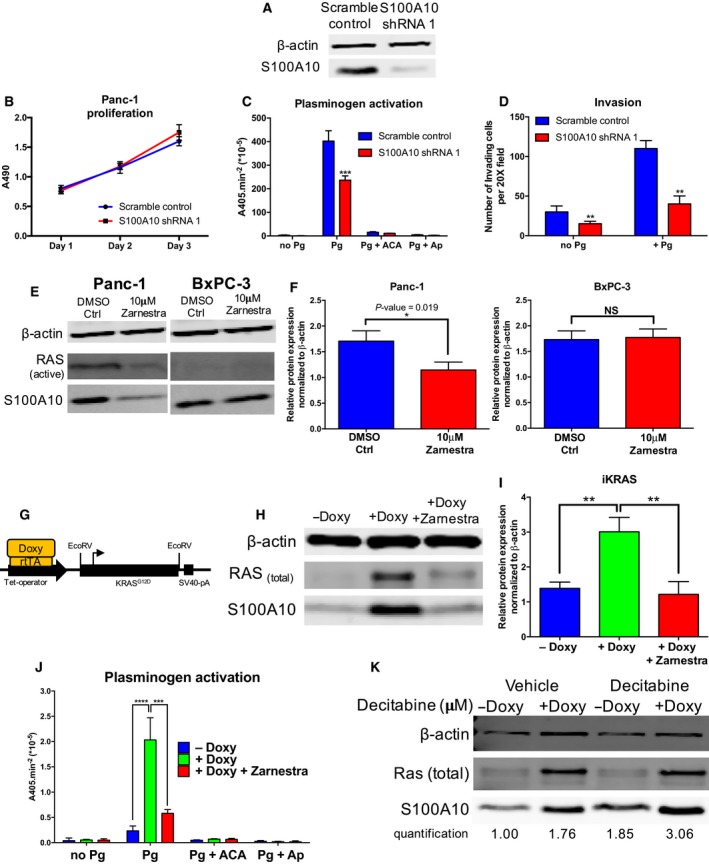
S100A10 modulates plasminogen activation and cellular invasiveness *in vitro* and is regulated by KRAS signaling. (A) Western blot of scramble control and S100A10‐depleted (S100A10 shRNA1) Panc‐1 cells. (B) Cells were equally seeded into a 96‐well plate and cell viability (MTS assay) was measured every day for three consecutive days. The absorbance of the MTS reagent at 490 nm is plotted for each time point. (C) Cells were incubated with 0.5 μm plasminogen, and plasmin activity was measured as the absorbance of the chromogenic plasmin substrate (S2251) at a wavelength of 405 nm. 5 × 10^3^ cells of scramble control and S100A10 shRNA1 Panc‐1 cells were seeded into 96‐well plates. Plasminogen activation (per 1 × 10^5^ cells) was then calculated under the following conditions: no plasminogen, with plasminogen, with the lysine analog ACA (100 mm) and the serine protease Ap (2.2 μm). ACA is a lysine analog that prevents plasminogen interaction with the carboxyl terminus. Ap is a serine protease pan‐inhibitor which quenches the generated plasmin confirming the ability of these cells to generate plasmin. (D) The matrigel Boyden chamber invasion assay assesses the ability of cells to invade through a Matrigel barrier (substitute for ECM) in response to a chemoattractant (10% FBS). Invasion assay of scramble control and S100A10 shRNA 1 Panc‐1 cells in the presence/absence of Pg. The results are represented as the number of invading cells per one field of view at 20× magnification. (E) Western blots of S100A10, active RAS, and β‐actin in Panc‐1 (a) and BxPC‐3 (c) treated with 10 μm of the farnesyltransferase inhibitor Zarnestra for 48 h. A Raf pulldown was performed to measure RAS activity. (F) Quantification of S100A10 protein expression normalized to β‐actin in DMSO‐ and Zarnestra‐treated Panc‐1 and BxPC‐3. (G) Genomic construct setup of the mouse iKRAS pancreatic cancer cells. rtTA is a reverse tetracycline transactivator and is required for doxycycline‐inducible expression of KRAS^G12D^. Western blot (H) and quantification (I) of S100A10 protein in iKRAS cells in the absence (−Doxy) or presence (+Doxy) of 1 μg·mL^−1^ doxycycline and Zarnestra (10 μm) for 4 days. (J) Plasminogen activation assay of IKRAS cells treated with doxycycline and Zarnestra). (K) Western blot analysis of iKRAS cells treated with doxycycline in the presence/absence of 10 μm decitabine for 72 h.

The data suggested a predictive role of *S100A10* mRNA expression and DNA methylation status as classifiers of patient outcome. However, the cellular mechanism by which S100A10 protein, as a plasminogen receptor, may contribute to the underlying pathology of PDAC remains unclear. The depletion of S100A10 using shRNA (Fig. 6A) in Panc‐1 cells did not affect their proliferation *in vitro* (Fig. 6B) but resulted in a 50% reduction in plasmin generation (i.e., plasminogen activation; Fig. 6C). Treatment with the pan‐plasminogen receptor inhibitor ɛ‐aminocaproic acid (ACA) completely abrogated plasmin generation indicating that plasmin is generation is primarily driven by plasminogen receptors of which S100A10 accounts for 50% (Fig. 6C). Subsequent assessment of cancer cell invasion revealed that S100A10 depletion reduced the ability of Panc‐1 cells (by 64%) to pass through the ECM‐dense matrigel even in the presence of exogenous plasminogen (+Pg) compared to scramble control cells (Fig. 6D). These findings inferred the role of S100A10 as a plasminogen receptor, which is an important regulator of plasmin generation and plasmin‐dependent invasiveness of pancreatic cancer cells.

### 3.8 S100A10 expression is regulated by oncogenic *KRAS* ^*G12D*^ in pancreatic cancer cells

We have previously demonstrated that RAS proteins, particularly HRAS, upregulate S100A10 expression in HEK293 cells (Madureira *et al*., 2012). Considering the direct involvement of oncogenic KRAS activity in PDAC pathobiology and the role of S100A10 in cellular proteolytic activity and invasiveness, we examined whether S100A10 is regulated via KRAS signaling. To address this, we utilized three cell lines representing three forms of *KRAS* expression, Bx‐PC3 (wild‐type *KRAS*), Panc‐1 (mutant *KRAS*,* KRAS* ^G12D^), and iKRAS (inducible *KRAS* ^G12D^). Treating BxPC‐3 and Panc‐1 cells with the farnesyltransferase inhibitor tipifarnib (Zarnestra) decreased S100A10 protein expression in the mutant *KRAS* cell line Panc‐1 but not in the wild‐type *KRAS* cell line BxPC3 (Fig. 6E,F). Similarly, ectopic expression of oncogenic *KRAS* ^G12D^ in *KRAS* wild‐type Bx‐PC3 (Fig. S12A) and HEK293 (Fig. S12B) cells also upregulated S100A10 protein expression. The iKRAS mouse cell line contains a doxycycline‐inducible *KRAS* ^G12D^ construct (Fig. 6G). The addition of 1 μg·mL^−1^ of doxycycline induced *KRAS* expression and a twofold increase in S100A10 protein expression which was abrogated by Zarnestra (Fig. 6H,I). *KRAS* induction dramatically increased plasminogen activation which was concomitant with S100A10 upregulation, while Zarnestra treatment abolished this activation (Fig. 6J). Considering the regulation of S100A10 by DNA methylation, we treated noninduced and induced cells with decitabine. Results revealed potentially independent effects of KRAS induction and promoter demethylation as the increase in S100A10 was higher in the presence of doxycycline and decitabine compared to either alone (Fig. 6K). These results revealed complex regulatory mechanism of S100A10‐ and S100A10‐mediated plasmin generation by oncogenic *KRAS*.

### 3.9 S100A10 is important for growth of pancreatic tumors

**Figure 7:**
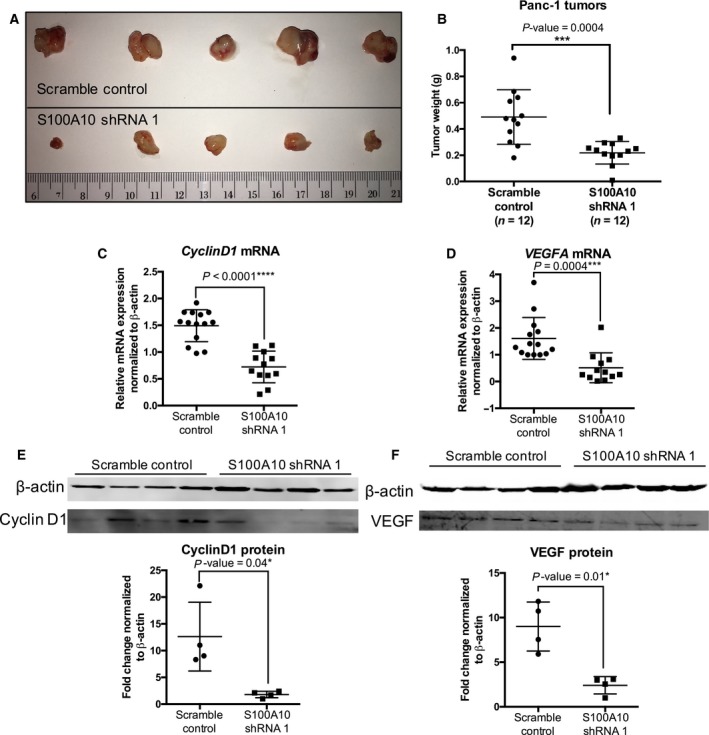
S100A10 depletion in Panc‐1 tumors reduces primary tumor size *in vivo*. 5 × 10^6^ scramble control and S100A10 shRNA 1 Panc‐1 cells were injected intraperitoneally into NOD/SCID mice. Representative images (A) and weight (B) of endpoint tumors (50 days postinjection). RT‐qPCR (C,D) and western blot (E,F) quantification of CCND1 (C,E) and VEGF (D,F).

To address whether S100A10 is implicated in *in vivo* PDAC tumorigenesis, we utilized a intraperitoneal model of PDAC. Schwarz *et al*. demonstrated that the intraperitoneal injection of Panc‐1 cells into immune‐deficient mice results in spontaneous homing of the Panc‐1 cells to the pancreas. This quasi‐orthotopic tumor development model shares many characteristics with human PDAC (Schwarz *et al*., 1999). After 8 weeks postintraperitoneal injection, juxta‐pancreatic tumors were extracted and weighed. Results show that tumors formed by S100A10‐depleted Panc‐1 cells (0.4913 g, CI: 0.3595 g–0.6230 g) were 2.24‐fold smaller than tumors formed by scramble control cells (0.2188 g, CI: 0.1644 g–0.2731 g; Fig. 7A,B). In an attempt to understand the difference in tumor size, we examined the expression of several genes involved in apoptosis (*BAD*,* BAX,* and *PUMA*), cell proliferation [cyclin D1 (*CCND1*)], metastasis (*MMP9*,* CDH1*,* CDH2,* and *VIM*), and angiogenesis [vascular endothelial growth factor (*VEGF*)] using RT‐qPCR (Fig. S11). The results showed that mRNA and subsequently protein levels of *CCND1* (Fig. 7C,E) and VEGF (Fig. 7D,F) were significantly lower in S100A10 shRNA 1 tumors compared to scramble control tumors. These results indicated that tumor cell S100A10 is important for *in vivo* growth of pancreatic tumors.

## 4 Discussion

Cancer progression is increasingly being attributed to aberrant expression of surface proteins that drive cancer invasion (Larkin and Aukim‐Hastie, 2011). These proteins are typically overexpressed in tumors and offer a unique opportunity for marker identification and potential therapeutic targeting. Expression of known driver genes (e.g., *TP53, CDK2NA, SMAD4*) in PDAC have linked to therapeutic outcome in patients. For instance, high protein expression of SMAD4 has been linked to better prognosis in PDAC patients postsurgical resection (Tascilar *et al*., 2001). However, novel genes have been also implicated in PDAC development and progression. During the early days of DNA microarrays, Iacobuzio‐Donahue *et al*. (2003) identified the gene encoding the plasminogen receptor *S100A10* as one of the upregulated genes in pancreatic tumors and cell lines compared to their normal counterparts. Many later studies aimed to further analyze differential gene expression using DNA microarrays and more recently RNA seq (Badea *et al*., 2008; Grützmann *et al*., 2004; Iacobuzio‐Donahue *et al*., 2003; Ishikawa *et al*., 2005; Logsdon *et al*., 2003; Pei *et al*., 2009; Segara *et al*., 2005). We analyzed these studies and demonstrated that *S100A10* mRNA is highly expressed in pancreatic tumors and cell lines (Figs S1 and S2). The question whether S100A10 protein was also upregulated was first addressed by a study by Sitek *et al*. (2009) who utilized mass spectrometry to identify 31 proteins (includes S100A10) that were overexpressed in pancreatic tumors. We herein performed an extensive semi‐automated quantification method of stained TMAs from 89 PDAC patients. The expression of S100A10 was found to be significantly low in pancreatic nonductal stroma and normal tissue and was unchanged even if the normal ducts or nonductal stroma were adjacent to PanINs or PDAC. There was, however, a significant but modest increase in expression in PanINs compared to normal ducts which was then dramatically elevated when PanINs progressed into PDAC (Fig. 3B). This presents the possibility that S100A10 upregulation by pancreatic tumors is a late event that appears to be unique to PDAC.

In addition to assessing S100A10 expression in pancreatic tissues, we addressed the novel predictive value of S100A10 in PDAC. *S100A10* mRNA expression and DNA methylation status were found to be predictive of long‐term OS and RFS in multiple patient cohorts (TCGA, ICGC; Moffit *et al*. cohort and Chen *et al*. cohort). We have developed a reliable ternary classification method through which we identified a low‐risk group of patients with very low *S100A10* mRNA levels or high *S100A10* methylation score. These patients had significantly longer survival and a lower probability of their cancers recurring. These results delineated, for the first time, the predictive role of S100A10 in PDAC. These finding are supported by other studies that addressed the predictive potential of S100A10 in various cancer models. For instance, Shang *et al*. (2013) revealed a correlation between positive S100A10 protein expression and poor tumor differentiation, disease stage, and poor OS in colorectal cancer patients. Li *et al*. (2017) demonstrated that, although S100A10 expression did not correlate with long‐term survival in gastric cancer patients, it did, however, correlate with lymph node positivity which is consistent with our multimodel fitting of OS and RFS (Table S8). Domoto *et al*. (2007) showed that S100A10 is an independent marker of survival in renal cell carcinoma while showing no correlation to tumor grade or stage of renal cell carcinoma patients. High *S100A10* mRNA and protein expression also predicted poorer OS in serous ovarian carcinoma (Lokman *et al*., 2016). These studies establish *S100A10* as a robust pan‐cancer biomarker of patient survivability and tumor progression.

The clinical significance of S100A10 in PDAC patients can be partly explained by its role in *in vitro* plasmin‐dependent proteolytic activity and invasiveness. As mentioned, plasminogen receptors are essential for the binding and the subsequent activation of the pro‐protease plasminogen into the active protease plasmin (Didiasova *et al*., 2014). Treatment with the lysine analog ε‐aminocaproic acid, which competes with plasminogen for receptor binding, completely abrogated plasminogen activation in Panc‐1 cells (Fig. 6C). Noteworthy, the significant reduction in invasion upon S100A10 depletion in the absence of plasminogen (−Pg) could be attributed to the plasminogen traces present in the serum used in these experiments (Loskutoff, 1978). This highlights the importance of plasminogen receptors, in general, in activating plasminogen in the presence of endogenous levels of plasminogen activators.

Epigenetic modulation of *S100A10* gene expression adds a layer of complexity to its regulation by *KRAS*. We have demonstrated that methylation of the ~ 400 bp promoter region of *S100A10* modulates its expression. Previous reports examining the 1q21 S100 genes revealed that regions upstream of the proximal 400 bp region were differentially methylated. The −600 to −745 region and −400 to −652 region were both found to be hypermethylated in human pituitary tumors (Dudley *et al*., 2008) and in medulloblastoma (Lindsey *et al*., 2007). It should be noted that although the TSS of exon 1 of S100A10 appears to be essential for gene regulation, the 97‐amino acid protein constitutes only exons 2 and 3. CpG islands often occur within gene promoters, and their methylation is linked to modulation of transcription. A potential CpG island spans the proximal promoter region, the untranslated region of exon 1 and part of intron 1 (Rice *et al*., 2000). This CpG island matches the stringent measures defined by Takai and Jones which necessitates that a region is considered a CpG island if it is longer than 500 bp with a G + C content equal to or > 55% and observed/expected CpG ratio is 0.65 or higher (Takai and Jones, 2002; Fig. S11C). The cg13249591 probe maps to the 5′ region of this CpG island, while the cg13445177 maps to its south shore. The cg13249591 contains two CpG sites whose methylation status was predictive of PDAC patient OS and RFS and was significantly demethylated in all three cell lines in response to decitabine.

Studies in the early 1990s demonstrated that KRAS increased levels of total (Buø *et al*., 1995) and receptor‐bound plasminogen activators (tPA and uPA) (Jankun *et al*., 1991) delineating the potential implication of the plasminogen activation system in KRAS‐mediated oncogenesis. Whether possible aberrant regulation of plasminogen receptors is implicated in PDAC has never been addressed. We demonstrated that the expression of the plasminogen receptor S100A10 was driven by oncogenic *KRAS* ^*G12D*^ which contributed to the enhancement of plasmin generation by pancreatic cancer cells (Fig. 6C,J; Fig. S14). This is supported by our recent findings which showed that S100A10 is driven by the RAS family of proteins in HEK293 cells via the RalGDS signaling arm. S100A10 enhanced Ras‐mediated plasminogen activation and was important for plasminogen‐dependent Ras‐induced invasion of HEK293 cells (Madureira *et al*., 2016). Notably, the ACA treatment of iKRAS cells abolished plasminogen activation in the absence and presence of induced *KRAS* ^*G12D*^ expression. As ACA blocks the interaction of plasminogen with plasminogen receptors but does not block the direct interaction of plasminogen with uPA or tPA, it is likely that the interaction of plasminogen with plasminogen receptors is the rate‐limiting step in plasmin generation by pancreatic cells. In addition, we have previously demonstrated that S100A10 colocalizes with uPA receptor (uPAR) at the cell surface of HT1080 fibrosarcoma (Choi *et al*., 2003) and Colo222 (Zhang *et al*., 2004) colorectal cancer cells to drive plasminogen activation. S100A10 is also capable of protecting plasmin from inactivation by α2‐antiplasmin (Kwon *et al*., 2005). Collectively, these studies strongly indicate that S100A10 is a central player in facilitating uPA‐mediated cleavage of plasminogen in *KRAS*‐transformed cancer cells (Fig. S14).

Considering the role of S100A10 in pancreatic cancer cell invasion *in vitro*, we addressed the role of S100A10 during *in vivo* tumorigenesis. The growth of Panc‐1 tumors in immunocompromised NOD/SCID mice was hindered upon depletion of S100A10 compared to the scramble control (Fig. 7A,B). This indicates that S100A10 depletion in these cells is sufficient to reduce tumor growth in the absence of tumor‐promoting immune cells. It should be noted that S100A10‐depleted Panc‐1 cells have similar proliferation rates *in vitro* (Fig. 6B) which suggests that the *in vivo* effects are likely mediated by the microenvironmental interactions with tumor cells. Our previous findings show that LLC cells yield dramatically smaller tumors in S100A10‐null mice compared to wild‐type mice and that both tumoral microenvironment and tumor‐associated macrophages were essential for sustaining tumor growth (Phipps *et al*., 2011). It remains unclear whether the reduced tumor growth is due to the plasminogen‐dependent function of S100A10 or a novel intracellular function related to apoptosis or proliferation. The latter is supported by evidence showing significant reduction in expression of VEGF and CCND1 in Panc‐1 tumors. Shan *et al*. (2013) recently demonstrated that the miR‐590‐5P directly binds 3′ UTR of S100A10 to inhibit its expression which was concomitant with downregulation of CCND1 in HepG2 hepatocellular carcinoma cells. In addition, Phipps *et al*. (2011) presented that S100A10‐deficient mice form a poorly vascularized environment for wild‐type S100A10 LLC cells based on CD31 staining. It is hence possible that tumor cell VEGF is required for adequate angiogenesis to occur. Our findings indicated that S100A10 contributes to tumor cell proliferation potentially via sustenance of CCND1 levels and to angiogenesis by maintaining VEGF production to ensure blood vessel development.

Biomarker discovery represents a direct translational path to clinical applications. S100A10 was found to be highly overexpressed in pancreatic tumors, regulated the fundamental process of cellular invasion, regulated by KRAS signaling and DNA methylation, and contributed to tumor growth. These findings delineate, for the first time, a comprehensive clinical and functional assessment of S100A10 in PDAC.

## 5 Conclusions

- S100A10 mRNA is highly expressed in pancreatic tumors and cell lines. S100A10 protein is also overexpressed in pancreatic tumors compared to adjacent nonductal stroma and normal ducts.
- S100A10 mRNA expression, copy number, and methylation status are predictive of overall and RFS in PDAC patients. In addition, S100A10 mRNA and lymph node status are linked predictors of overall and RFS.
- S100A10 expression is regulated by methylation at several promoter CpG sites. It is also regulated by oncogenic *KRAS* ^*G12D*^ and serves as a plasminogen receptor to mediate cancer cell invasion *in vitro*.
- S100A10 is important for *in vivo* growth of pancreatic tumors and modulates expression of VEGF and CCND1.

## Author contributions

MB and DMW involved in conception and design. MB, AS, ICGW, WYH, and DMW involved in development of methodology. MB, AS, HL, GJR, ICGW, AU, WYH, and DMW involved in acquisition of data (provided animals, acquired and managed patients, provided facilities, etc.). MB, HL, GJR, ICGW, HG, and DMW performed analysis and interpretation of data (e.g., statistical analysis, biostatistics, computational analysis). MB, DMW, HG, ICGW, AS, AU, and WYH performed writing, review, and/or revision of the manuscript. MB and HG provided administrative, technical, or material support (i.e., reporting or organizing data). DMW involved in study supervision. WYH involved in actively selecting patient to enroll in the study, collection, and review of clinical data; in obtaining patient consent for the study, communicate with multidisciplinary team members and the hospital and research staff to obtain the tissue sample according to the Hospital IRB/HIPPA; and in acquisition of appropriate tissue samples.

## Supporting information


**Fig. S1**. S100A10 mRNA is over‐expressed in pancreatic TCGA tumors and CCLE cell lines.
**Fig. S2**. S100A10 mRNA is overexpressed in pancreatic tumors compared to normal pancreatic tissue.
**Fig. S3.** Representative images of S100A10 staining in normal ducts and cancerous lesions.
**Fig. S4.** The three cut‐offs of S100A10 mRNA.
**Fig. S5.** Correlation of *S100A10* mRNA expression, linear copy number and copy number status with overall and RFS.
**Fig. S6.** The β values of probes that were not differentially‐methylated and/or did not negatively correlate with *S100A10* mRNA expression.
**Fig. S7**. Kaplan Meier survival analyses of OS based on β values of the remaining four probes in the TCGA PDAC cohort.
**Fig. S8**. Kaplan Meier survival analyses of RFS based on β values of the remaining four probes in the TCGA PDAC cohort.
**Fig. S9**. Kaplan Meier analyses of CpG islands corresponding to probes cg13249591 and cg13445177 using median and optimal cut‐offs.
**Fig. S10**. The β values of the probes cg13445177 and cg13249591 do not positively correlate with mRNA expression of *de novo* methyltransferases.
**Fig. S11**. S100A10 promoter methylation.
**Fig. S12**. Effect of oncogenic KRAS^G12D^ on S100A10 expression in WT‐KRAS cells.
**Fig. S13.** RT‐qPCR of several genes in scramble control and S100A10‐shRNA 1 Panc‐1 tumors.
**Fig. S14**. Schematic representation of KRAS^G12D^‐ and methylation‐mediated regulation of S100A10‐dependent plasminogen activation.
**Table S1**. Calculation scheme of the *H*‐score.
**Table S2**. Higher S100A10 mRNA, higher copy number and low‐methylation scores correlate with lower short‐term survival.
**Table S3.** Multiple comparisons of OS and RFS using the mRNA Ternary classifier.
**Table S4.** Univariate cox regression analysis of OS of the TCGA PDAC cohort.
**Table S5**. Multivariate cox regression analysis of OS of the TCGA PDAC cohort.
**Table S6**. Univariate cox regression analysis of RFS of the TCGA PDAC cohort.
**Table S7.** Multivariate cox regression analysis of RFS of the TCGA PDAC cohort.
**Table S8.** Final co‐variate models of OS and RFS in the TCGA PDAC cohort.
**Table S9**. The location and target sequence of 15 methylation probes associated with S100A10. **Table S10.** Multiple comparisons of OS and RFS using the mRNA Ternary classifier.
**Table S11**. List of primer sequences used in RT‐qPCR and pyrosequencing as well as dsDNA oligo used for S100A10 shRNA.
