# Supplementary material for: S100A10, a novel biomarker in pancreatic ductal adenocarcinoma
Source: Mol Oncol. 2018 Sep 21;12(11):1895–916. doi: 10.1002/1878-0261.12356 (PMC6210040; doi:10.1002/1878-0261.12356)
Supplement: Supplementary file 1 — Fig. S1. S100A10 mRNA is over‐expressed in pancreatic TCGA tumors and CCLE cell lines. Fig. S2. S100A10 mRNA is overexpressed in pancreatic tumors compared to normal pancreatic tissue. Fig. S3. Representative images of S100A10 staining in normal ducts and cancerous lesions. Fig. S4. The three cut‐offs of S100A10 mRNA. Fig. S5. Correlation of S100A10 mRNA expression, linear copy number and copy number status with overall and RFS. Fig. S6. The β values of probes that were not differentially‐methylated and/or did not negatively correlate with S100A10 mRNA expression. Fig. S7. Kaplan Meier survival analyses of OS based on β values of the remaining four probes in the TCGA PDAC cohort. Fig. S8. Kaplan Meier survival analyses of RFS based on β values of the remaining four probes in the TCGA PDAC cohort. Fig. S9. Kaplan Meier analyses of CpG islands corresponding to probes cg13249591 and cg13445177 using median and optimal cut‐offs. Fig. S10. The β values of the probes cg13445177 and cg13249591 do not positively correlate with mRNA expression of de novo methyltransferases. Fig. S11. S100A10 promoter methylation. Fig. S12. Effect of oncogenic KRASG12D on S100A10 expression in WT‐KRAS cells. Fig. S13. RT‐qPCR of several genes in scramble control and S100A10‐shRNA 1 Panc‐1 tumors. Fig. S14. Schematic representation of KRASG12D‐ and methylation‐mediated regulation of S100A10‐dependent plasminogen activation. Table S1. Calculation scheme of the H‐score. Table S2. Higher S100A10 mRNA, higher copy number and low‐methylation scores correlate with lower short‐term survival. Table S3. Multiple comparisons of OS and RFS using the mRNA Ternary classifier. Table S4. Univariate cox regression analysis of OS of the TCGA PDAC cohort. Table S5. Multivariate cox regression analysis of OS of the TCGA PDAC cohort. Table S6. Univariate cox regression analysis of RFS of the TCGA PDAC cohort. Table S7. Multivariate cox regression analysis of RFS of the TCGA PDAC cohort. Table S8. Final co‐variate [file MOL2-12-1895-s001.pdf]

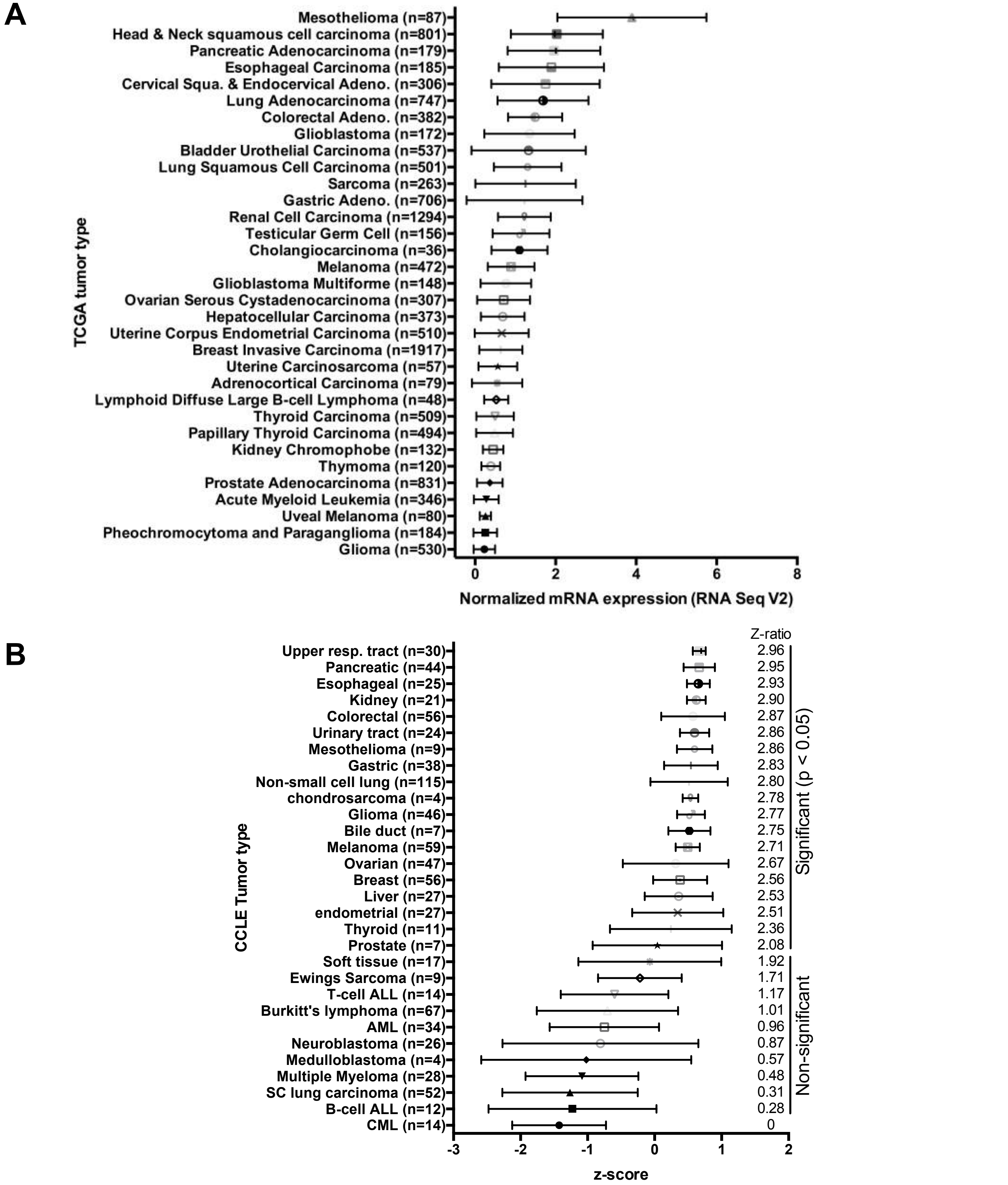

Supplemental Figure 1. S100A10 mRNA is over-expressed in pancreatic TCGA tumors and CCLE cell lines. (A) S100A10 REVs (RNA Seq V2 RSEM) were extracted from Cbioportal and normalized by dividing by the mean REV of the 33 TCGA tumor types. (B) Z-scores of S100A10 of the 930 CCLE cell lines were extracted from Cbioportal. Z-ratios were used to determine significance with respect to CML (control). A z-ratio of 1.96 is equivalent to a p-value of 0.05.

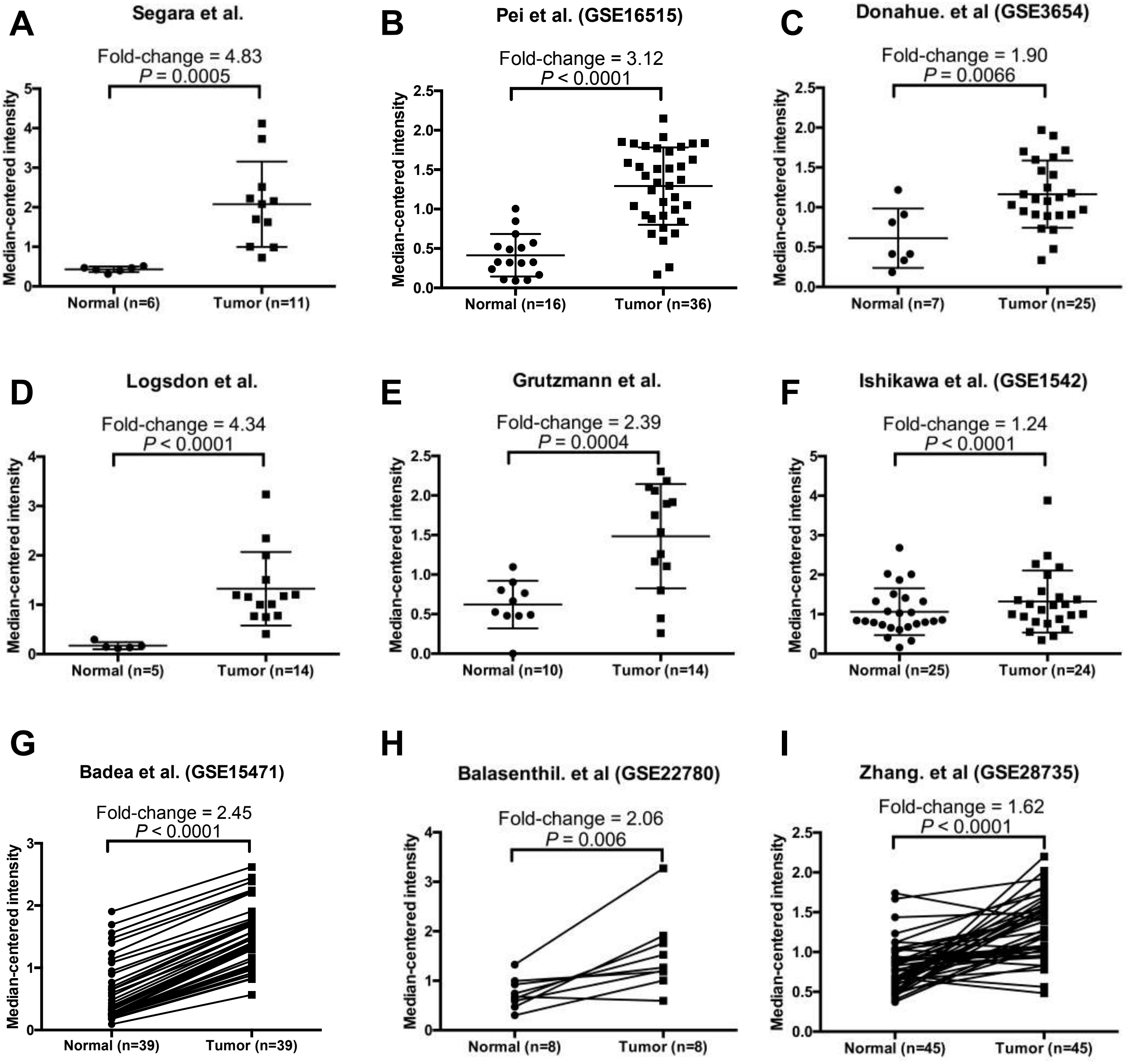

Supplemental Figure 2. S100A10 mRNA is overexpressed in pancreatic tumors compared to normal pancreatic tissue. Gene expression from 9 publicly available gene expression datasets were extracted from Oncomine (A-D, F, G, I) and gene expression omnibus (GEO) (E, H). The datasets compare gene expression in normal vs. tumor from pancreatic cancer patients. Badea *et al*. (G), Balasenthil *et al*. (H) and Zheng *et al*. (I) represent matched samples of pancreatic tumors and corresponding adjacent normal tissue. Significance was determined using unpaired (A-F) or paired (G-I) t-tests. Significance was determined based on a p-value of 0.05. Data are represented as means ± SD.

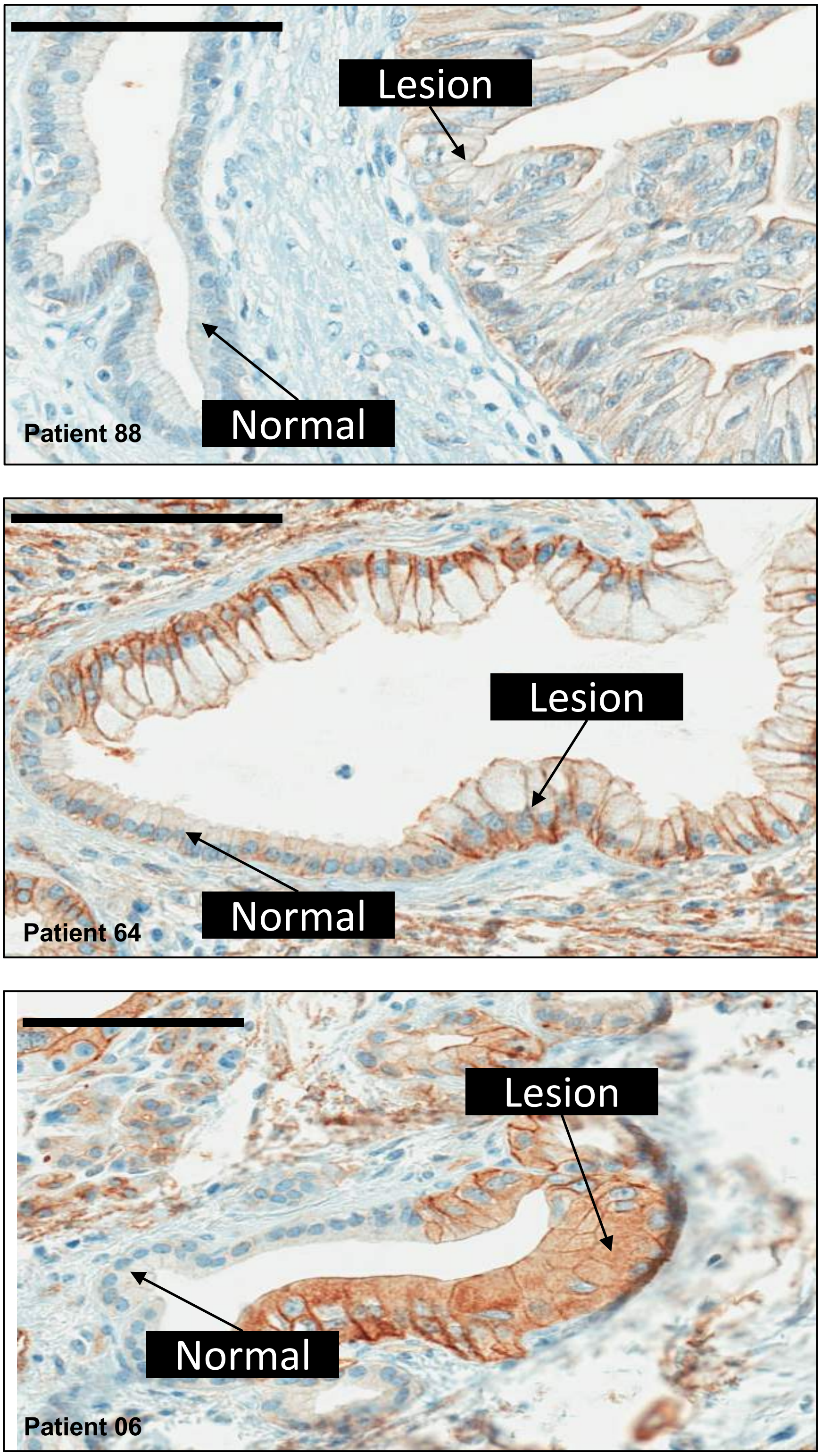

Supplemental Figure 3. Representative images of S100A10 staining in normal ducts and cancerous lesions. Images represent three patient samples showing the upregulation of S100A10 (IHC) in tumor ducts/lesions compared to normal ducts. Scale bars, 100 µm.

Score	Staining intensity
**0**	Negative
**1**	Low positive
**2**	Positive
**3**	High Positive

H-score =

0 x percentage contribution of negative pixels
1 x percentage contribution of low positive pixels
2 x percentage contribution of positive pixels
3 x percentage contribution of highly positive pixels

Supplemental Table 1. Calculation scheme of the H-score. The score represents both the intensity and number of DAB-positive pixels in stained sample.

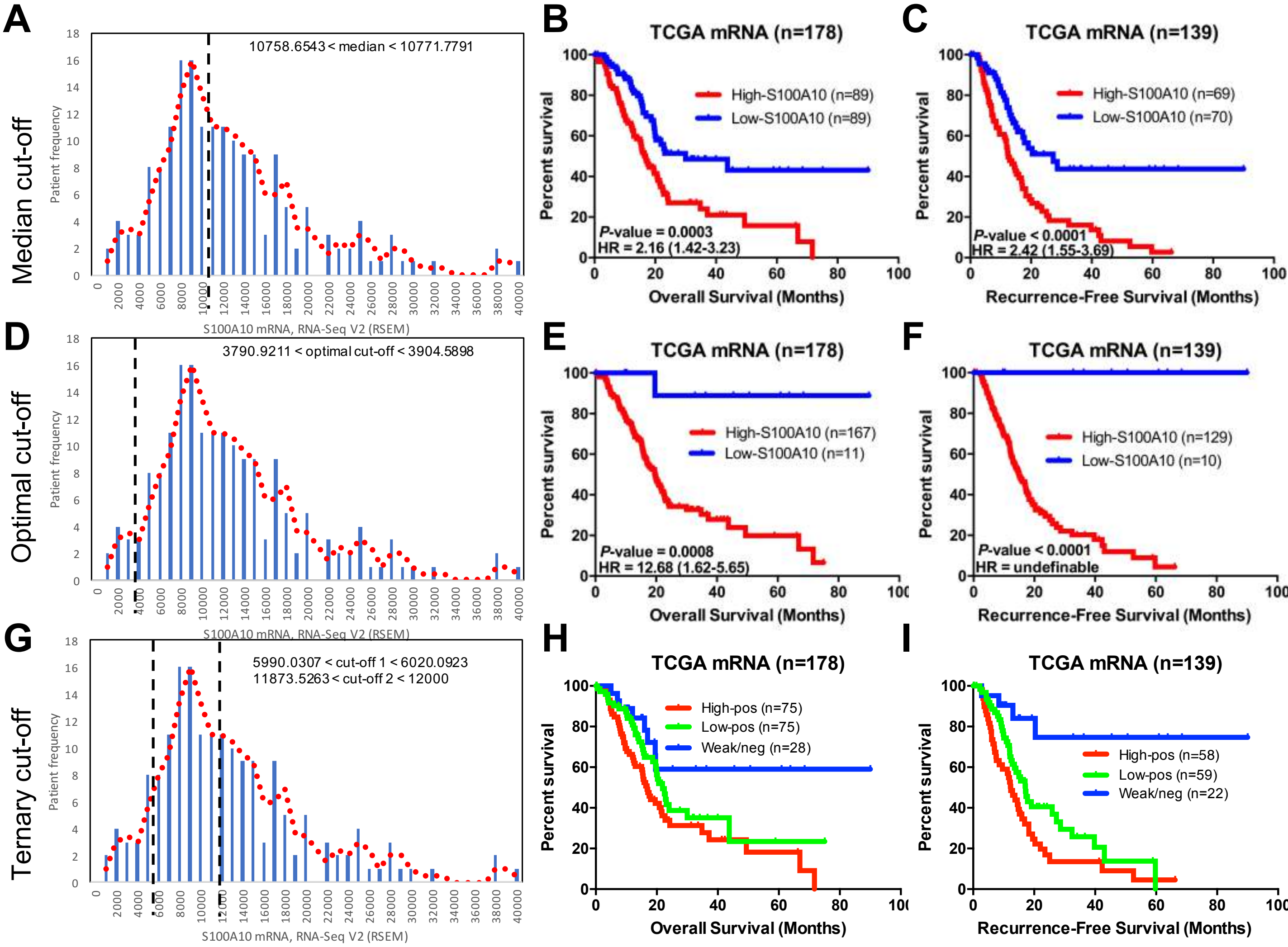

Supplemental Figure 4. The three cut-offs of S100A10 mRNA. S100A10 REVs of the TCGA PDAC cohort follow a relatively normal Gaussian distribution. The three cut-off system is based on the median expression value (A), optimal expression value (D), or a ternary expression classifier (CG). The median cut-off is based on the median REV. In the case of even patient number, the median REV was considered high or low based on closeness of expression. Optimal cut-offs were determined using the cut off finder database based on the lowest p-value and highest hazard ratio possible. Source: (http://molpath.charite.de/cutoff/) Budczies *et al*. (2012), PLoS ONE 7(12): e51862. The Ternary cut-off was derived from bin frequency of REVs. This identified two cut-off REVs resulting in three expression groups. Kaplan Meier analyses of overall (B, E, H) and recurrence-free survival (C, F, I) based on the median cut-off (B, C), the optimal cut-off (E, F) and the ternary cut-off (H, I).

S100A10 genomic profile	Overall Survival (OS)			Recurrence-free Survival (RFS)		
	1-yr survival	3-yr survival	5-yr survival	1-yr survival	3-yr survival	5-yr survival
**Low mRNA**	69.66% (62/89)	13.48% (12/89)	5.62% (5/89)	58.57% (41/70)	14.29% (10/70)	5.71% (4/70)
**High mRNA**	59.55% (53/89)	8.99% (8/89)	3.37% (3/89)	49.28% (34/69)	10.14% (7/69)	1.45% (1/69)
**High copy number**	65.91% (58/88)	10.23% (9/88)	3.41% (3/88)	48.57% (34/70)	8.570% (6/70)	1.43% (1/70)
**Low copy number**	62.5% (55/88)	12.5% (11/88)	5.68% (5/88)	57.97% (40/69)	15.94% (11/69)	5.80% (4/69)
**High methylation score**	65.17% (58/89)	6.74% (6/89)	2.25% (2/89)	55.71% (39/70)	17.14% (12/70)	5.71% (4/70)
**Low methylation score**	64.04% (57/89)	15.73% (14/89)	6.74% (6/89)	52.17% (36/69)	7.25% (5/69)	1.45% (1/69)

Supplemental Table 2. Higher S100A10 mRNA, higher copy number and low-methylation scores correlate with lower short-term survival. The percentages represent the percentages of patients alive or recurrence-free at one, three and five year time point during this study. These percentages also represent the likelihoods of being alive or recurrence-free after one, three and five years if the TCGA cohort is similar to the entire population PDAC cohort.

S100A10 mRNA									
	weak neg vs. high-pos			weak neg vs. low-pos			low-pos vs. high pos		
Study	HR	p-value	C.I.	HR	p-value	C.I.	HR	p-value	C.I.
TCGA OS	2.84	0.0039	1.310 to 4.000	1.855	0.1056	0.8974 to 3.317	1.457	0.0856	0.9503 to 2.236
TCGA RFS	6.668	< 0.0001	2.067 to 6.833	4.742	0.0008	1.640 to 5.919	1.597	0.0353	1.036 to 2.545
GSE57495 OS	2.929	0.0402	1.047 to 5.788	3.28	0.02	1.180 to 6.058	0.9491	0.8716	0.5021 to 1.792
GSE71729 OS	3.365	0.0026	1.454 to 5.182	4.373	< 0.0001	1.926 to 6.163	0.7575	0.2055	0.4734 to 1.158
ICGC OS	3.715	0.0073	1.296 to 5.112	2.692	0.0495	1.007 to 4.458	1.429	0.1297	0.9028 to 2.350

Supplemental Table 3. Multiple comparisons of OS and RFS using the mRNA Ternary classifier. Multiple comparisons of S100A10 mRNA survival functions were performed on the TCGA, Chen *et al*. (GSE57495, n=63), Moffitt *et al*. (GSE71729, n=125) and ICGC (international cancer genome consortium, n=133). *P*-values were adjusted to the Bonferroni-corrected threshold. Adjusted *p*-value is *p*-value/K = 0.017 where K=3 and represents the number of comparisons made.

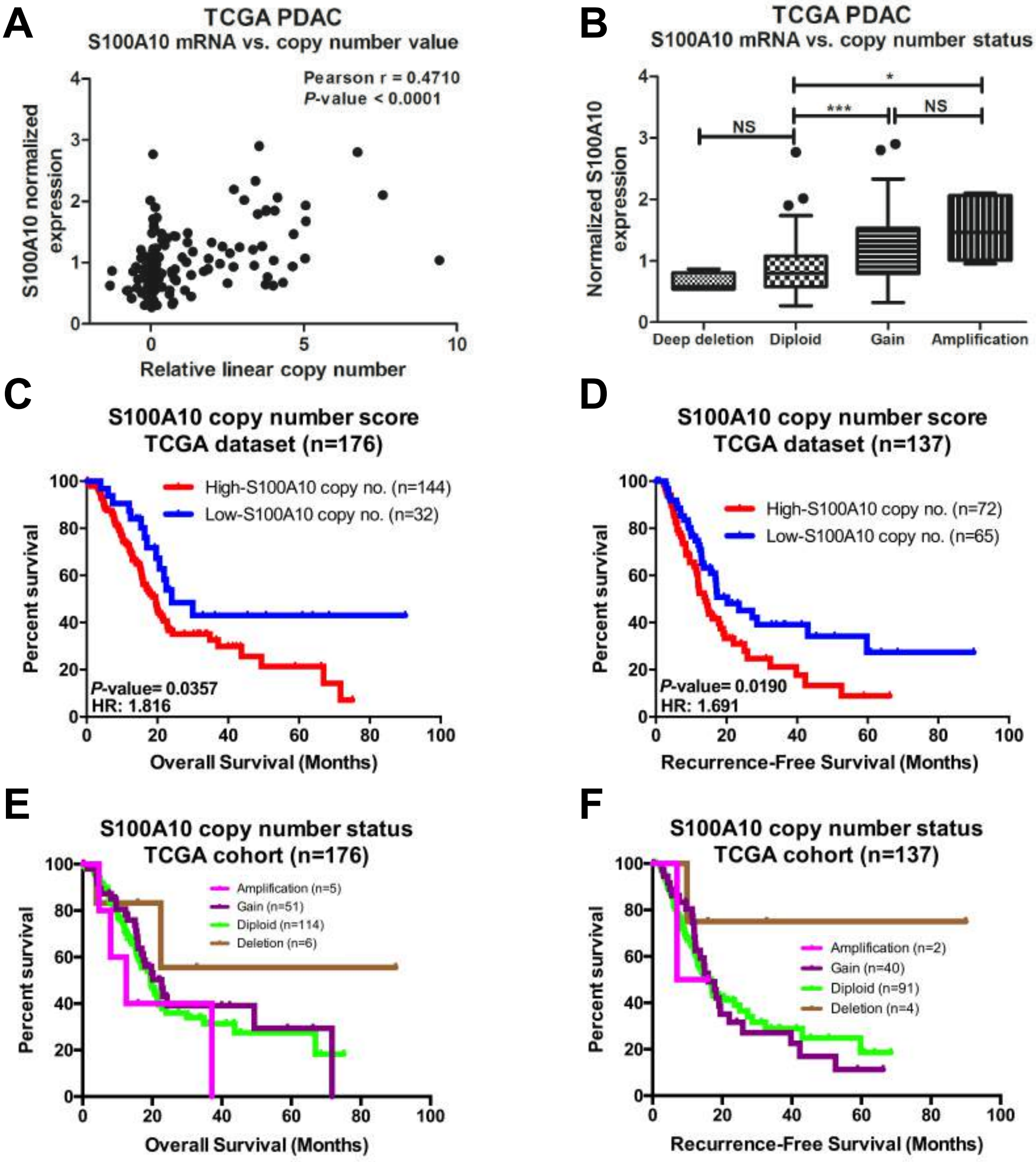

Supplemental Figure 5. Correlation of *S100A10* mRNA expression, linear copy number and copy number status with overall and recurrence-free survival. Pearson correlation analysis of S100A10 mRNA (expression values normalized to average) with (A) relative linear copy number and (B) copy number status. Kaplan Meier analysis of overall survival of TCGA PDAC patients in relation to S100A10 copy number score based on an optimal cut-off of (C) OS and (D) RFS. Kaplan Meier analysis of (E) OS and (F) RFS based on copy number status of *S100A10*. Gain and amplification are based on the Cbioportal definition where gain represents a low-level increase in copy number while amplification represents a high-level of increase.

Univariate analysis									
**Variable**	coef	exp(coef)	se(coef)	z	Pr(> |z|)	exp(-coef)	lower .95	upper .95	Significant (Y/N)
**S100A10 mRNA**	0.58	1.79	0.16	3.55	0.00038	0.56	1.3	2.46	Y
**Gender**									
Female (n=80)	0	1	-	-	-	-	-	-	
Male (n=97)	-0.22	0.81	0.21	-1.03	0.3	1.24	0.53	1.21	N
**Race**									
White (n=156)	0	1	-	-	-	-	-	-	
Asian (n=11)	-0.23	0.79	0.46	-0.5	0.62	1.26	0.32	1.97	N
Black/African American (n=6)	-0.04	0.96	0.51	-0.08	0.94	1.04	0.35	2.62	N
**age**	0.03	1.03	0.01	2.65	0.008	0.97	1.01	1.05	Y
**Grade**									
Grade I (n=31)	0	1	-	-	-	-	-	-	
Grade II (n=95)	0.69	2	0.34	2.04	0.04	0.5	1.03	3.88	Y
Grade III (n=47)	0.93	2.55	0.36	2.61	0.01	0.39	1.26	5.14	Y
Grade IV (n=2)	0.51	1.67	1.05	0.49	0.62	0.6	0.21	13.05	N
**Tumor dimension** (n=164)	0	1	0.06	0.09	0.93	1	0.9	1.12	N
**Lymph node involvement**									
N0 (negative, n=49)	0	1	-	-	-	-	-	-	
N1 (positive, n=123)	0.74	2.09	0.26	2.78	0.01	0.48	1.24	3.51	Y
**Metastasis**									
M0 (no mets, n=79)	0	1	-	-	-	-	-	-	
M1 (mets, n=4)	-0.07	0.94	0.73	-0.09	0.93	1.07	0.23	3.88	N
**Stage**									
Stage I (n=21)	0	1	-	-	-	-	-	-	
Stage II (n=146)	0.85	2.33	0.40	2.12	0.03	0.43	1.07	5.08	Y
Stage III (n=3)	0.23	1.26	1.07	0.22	0.83	0.79	0.15	10.32	N
Stage IV (n=5)	0.77	2.15	0.81	0.95	0.34	0.46	0.44	10.51	N
**Smoking** (n=56)	0	1	0.01	-0.05	0.96	1	0.98	1.02	N
**alcohol consumption (Yes, n=**101**/No, n=**64)	-0.1	0.91	0.22	-0.44	0.66	1.1	0.58	1.41	N

Supplemental Table 4. Univariate cox regression analysis of overall survival (OS) of the TCGA PDAC cohort. Abbreviations are as follows: Coef: beta coefficients, exp(coef): exponential of the coefficient, se(coef): standard error of the coefficient, z: Z statistics to test coefficient =0, Pr(> |z|): P-value based on the Wald test to test coefficient =0, Exp(-coef): exponential of the negative coefficient, Lower .95 and upper .95: the lower and upper limits for the 95% CI for exp(coef). See methods for additional details.

Multivariate analysis									
**Variable**	coef	exp(coef)	se(coef)	z	Pr(> |z|)	exp(-coef)	lower .95	upper .95	Significant (Y/N)
**S100A10 mRNA**	0.46	1.58	0.2	2.27	0.02	0.63	1.07	2.35	Y
**Gender**									
Female (n=80)	0	1	-	-	-	-	-	-	
Male (n=97)	-0.35	0.71	0.23	-1.5	0.13	1.42	0.45	1.11	N
**Race**									
White (n=156)	0	1	-	-	-	-	-	-	
Asian (n=11)	-1.81	0.16	1.61	-1.12	0.26	6.09	0.01	3.83	N
Black/African American (n=6)	0.51	1.66	0.54	0.93	0.35	0.6	0.57	4.8	N
**age**	0.02	1.02	0.01	1.33	0.18	0.99	0.99	1.04	N
**Grade**									
Grade I (n=31)	0	1	-	-	-	-	-	-	
Grade II (n=95)	0.29	1.34	0.39	0.74	0.46	0.75	0.62	2.87	N
Grade III (n=47)	0.4	1.49	0.41	0.98	0.33	0.67	0.67	3.29	N
Grade IV (n=2)	-0.17	0.85	1.07	-0.16	0.88	1.18	0.1	6.91	N
**Tumor dimension** (n=164)	0.06	1.06	0.07	0.8	0.42	0.94	0.92	1.22	N
**Lymph node involvement**									
N0 (negative, n=49)	0	1	-	-	-	-	-	-	
N1 (positive, n=123)	0.78	2.18	0.36	2.18	0.03	0.46	1.08	4.39	Y
**Metastasis**									
M0 (no mets, n=79)	0	1	-	-	-	-	-	-	
M1 (mets, n=4)	-0.09	0.92	1.27	-0.07	0.95	1.09	0.08	11.14	N
**Stage**									
Stage I (n=21)	0	1	-	-	-	-	-	-	
Stage II (n=146)	-0.43	0.65	0.56	-0.77	0.44	1.54	0.22	1.96	N
Stage III (n=3)	-0.49	0.61	1.09	-0.45	0.65	1.63	0.07	5.23	N
Stage IV (n=5)	N/A	N/A	N/A	N/A	N/A	N/A	N/A	N/A	

Supplemental Table 5. Multivariate cox regression analysis of overall survival (OS) of the TCGA PDAC cohort. See methods for additional details.

Univariate analysis									
**Variable**	coef	exp(coef)	se(coef)	z	Pr(> |z|)	exp(-coef)	lower .95	upper .95	Significant (Y/N)
**S100A10 mRNA**	0.75	2.12	0.17	4.47	0	0.47	1.52	2.94	Y
**Gender**									
Female (n=63)	0	1							
Male (n=78)	-0.17	0.84	0.22	-0.77	0.44	1.18	0.55	1.3	N
**Race**									
White (n=123)	0	1	-	-	-	-	-	-	N
Asian (n=8)	-0.02	0.98	0.46	-0.05	0.96	1.02	0.39	2.43	N
Black/African American (n=5)	0.26	1.3	0.52	0.51	0.61	0.77	0.47	3.57	N
**age**	0.02	1.02	0.01	1.8	0.07	0.98	1	1.04	N
**Grade**									
Grade I (n=28)	0	1	-	-	-	-	-	-	
Grade II (n=72)	0.76	2.14	0.35	2.19	0.029	0.47	1.08	4.23	Y
Grade III (n=37)	1.19	3.29	0.36	3.27	0.001	0.3	1.61	6.71	Y
Grade IV (n=2)	0.35	1.42	1.05	0.33	0.74	0.71	0.18	11.11	N
**Tumor dimension** (n=127)	-0.02	0.98	0.06	-0.33	0.74	1.02	0.87	1.1	N
**Lymph node involvement**									
N0 (negative, n=43)	0	1	-	-	-	-	-	-	
N1 (positive, n=95)	0.59	1.80	0.25	2.36	0.018	0.56	1.10	0.59	Y
**Metastasis**									
M0 (no mets, n=71)	0	1	-	-	-	-	-	-	N
M1 (mets, n=3)	-0.13	0.88	0.72	-0.18	0.86	1.14	0.21	3.63	N
**Stage**									
Stage I (n=21)	0	1	-	-	-	-	-	-	
Stage II (n=114)	1.02	2.77	0.40	2.53	0.01	0.36	1.26	6.12	Y
Stage III (n=4)	0.88	2.41	1.08	0.82	0.41	0.42	0.29	19.82	N
Stage IV (n=3)	1.03	2.80	0.81	1.27	0.21	0.36	0.57	13.73	N
**Smoking** (n=43)	0	1	0.01	0.05	0.96	1	0.98	1.02	N
**alcohol consumption (Yes,** n=82**/No,** n=49**)**	-0.2	0.81	0.24	-0.85	0.4	1.23	0.51	1.31	N

Supplemental Table 6. Univariate cox regression analysis of Recurrence-free survival (RFS) of the TCGA PDAC cohort. See methods for additional details.

Multivariate analysis									
**Variable**	coef	exp(coef)	se(coef)	z	Pr(> |z|)	exp(-coef)	lower .95	upper .95	Significant (Y/N)
**S100A10 mRNA**	0.54	1.71	0.22	2.49	0.01	0.58	1.12	2.61	Y
**Gender**									
female	0	1	-	-	-	-	-	-	
male	-0.27	0.76	0.26	-1.03	0.3	1.31	0.46	1.27	N
**Race**									
White	0	1	-	-	-	-	-	-	
Asian	-16.43	0	2767.98	-0.01	1	0.39	0	Inf	N
Black/African american	0.89	2.44	0.64	1.39	0.16	0.41	0.7	8.54	N
**age**	0.01	1.01	0.01	0.48	0.63	0.99	0.98	1.03	N
**Grade**									
Grade I (n=32)	0	1							
Grade II (n=97)	0	1	0.42	0	1	1	0.44	2.25	N
Grade III (n=50)	0.34	1.41	0.44	0.79	0.43	0.71	0.6	3.31	N
Grade IV (n=5)	-0.73	0.48	1.08	-0.68	0.49	2.09	0.06	3.94	N
**Tumor dimension**	0.03	1.03	0.08	0.35	0.73	0.97	0.88	1.2	N
**Lymph node involvement**									
N1 (positive, n=129)	0	1	-	-	-	-	-	-	
N0 (negative, n=50)	-0.68	0.51	0.34	-1.97	0.05	1.97	0.26	1	Y
**Metastasis**									
M0 (no mets, n=84)	0	1	-	-	-	-	-	-	
M1 (no mets, n=5)	-1.01	0.37	1.3	-0.78	0.44	2.74	0.03	4.65	N
**Stage**									
Stage I (n=21)	0	1	-	-	-	-	-	-	
Stage II (n=114)	-0.46	0.63	1.1	-0.42	0.67	1.59	0.07	5.44	N
Stage III (n=4)	0.46	1.59	1.10	0.42	0.68	0.63	0.18	13.7	N
Stage IV (n=3)	N/A	N/A	N/A	N/A	N/A	N/A	N/A	N/A	

Supplemental Table 7. Multivariate cox regression analysis of Recurrence-free survival (RFS) of the TCGA PDAC cohort. See methods for additional details.

OS								
**Variable**	coef	exp(coef)	se(coef)	z	Pr(> |z|)	exp(-coef)	lower .95	upper .95
**S100A10 mRNA**	0.43	1.54	0.19	2.31	0.02	0.65	1.07	2.21
**Lymph node (N1)**	0.66	1.93	0.27	2.48	0.01	1.93	1.15	3.24
**age**	0.02	1.02	0.01	2.08	0.04	0.98	1	1.04

RFS								
**Variable**	coef	exp(coef)	se(coef)	z	Pr(> |z|)	exp(-coef)	lower .95	upper .95
**S100A10 mRNA**	0.64	1.89	0.19	3.44	0	0.53	1.32	2.72
**Lymph node (N1)**	0.43	1.54	0.25	1.71	0.09	0.65	0.94	2.53

Supplemental Table 8. Final co-variate models of OS and RFS in the TCGA PDAC cohort. Two and three variables were considered significant based on multivariate regression models of OS and RFS respectively. These models calculate hazard ratios based on the most significant variables in predicting OS and RFS.

Name	Strand	UCSC Ref	Genomic sequence (5’-3’)
cg04989070	-	5'UTR	**CG**AGAAAATAGCAAGTGTTAGAAGAGAAGGAGCACAGTCATGTCATTCTG
cg05368119	+	5’UTR/Body	**CG**TGTTCCATTTGAGATGGCATTTTGGTGTGGTC**CG**TTGAAGCCTATTAA
cg06698332	-	TSS1500	**CG**AGGAGTTGGTAAGCATCCCCTAGGAAACACTTAGGTTTTCTCTAAATT
cg06786599	-	5'UTR	**CGCG**CCCCTCCTGGGTAGTCCCCAGGCC**CG**GACCTGCTGCC**CG**GGGAAAA
**cg13249591**	+	TSS200	**CG**GTTTGGCTTGTCAGCACCCAGGGG**CG**TCACAAACCCTTTGTTGAACAG
**cg13445177**	-	TSS1500	**CG**CC**CG**GCCAGTTTTTAACACTATTAGCCACACTGAAACTGAACTATTGA
cg17711091	-	5'UTR	**CG**AATCCCTCCTACA**CG**CCCCTGCCCTGGCTGG**CG**TGTTTCTTGGTGGGT
cg18348690	-	TSS1500	**CG**GAAAGTAATAGCTGAAATCCAAGTTGGGTTTTCCTGGCAACAGCCAAT
cg18892537	-	5'UTR	**CG**GCTGGTGGGGAATC**CG**CTGCTCAGTGCTC**CG**GGCCACACCCAAA**CG**AG
cg20167074	-	TSS1500	**CGCG**CC**CG**GCCAGTTTTTAACACTATTAGCCACACTGAAACTGAACTATT
cg20994097	+	TSS1500	**CG**TTAGATAAGCAAACAATTAAAATCCAGTGTGCTTAGTGAATGGTAGAG
cg24594295	-	TSS200	**CG**GACCTCCTAGGGCTAATCTGATAGTGCCTCTGAGGT**CG**ATAGGACTCC
cg25848158	-	TSS200	**CG**C**CG**AGACCCCCAGA**CG**GACCTCCTAGGGCTAATCTGATAGTGCCTCTG
cg26230275	-	5'UTR	**CG**CTCCAAGCCAGGCCAGCACAGGGGAGCCCTAAGCCAGATTCTGGGATG
cg16658496	+	Exon1;5'UTR	**CG**CCCTGCCCT**CG**CTCC**CG**GACC**CG**CCT**CG**CAGAGGCCT**CG**CC**CG**CCCCA

Supplemental Table 9. The location and target sequence of 15 methylation probes associated with S100A10. All 15 probe sites were extracted from the Illumina Human Methylation450 v1.2 (https://support.illumina.com) and CpG sites identified in the genomic sequence complementary to each probe. T_cSS: transcription start site, T_LSS: translation start site, TSS1500: region between 200bp and 1500bp upstream of T_cSS, TSS200: region 200bp upstream of T_cSS, 5’UTR: 5’ untranslated region. Each probe covers one or more CpG sites and may overlap with other probes.

S100A10 methylation OS TCGA									
	high vs. low			high vs. intermediate			low vs. intermediate		
Probe	HR	p-value	C.I.	HR	p-value	C.I.	HR	p-value	C.I.
cg13249591	1.702	0.094	0.9280 to 2.876	1.53	0.1936	0.8210 to 2.697	1.203	0.4002	0.7814 to 1.878
cg13445177	1.848	0.0613	0.9831 to 3.100	2.041	0.0298	1.076 to 3.403	0.9533	0.8276	0.6155 to 1.473
cg18348690	1.668	0.1338	0.8744 to 2.861	1.399	0.3442	0.7173 to 2.599	1.268	0.2842	0.8221 to 1.954
cg26230275	1.472	0.2664	0.7660 to 2.660	1.704	0.1244	0.8797 to 2.936	0.8486	0.4559	0.5488 to 1.308
cg20994097	1.577	0.2641	0.7358 to 3.350	1.321	0.5103	0.5955 to 2.866	1.154	0.6117	0.6591 to 2.050
cg06698332	1.535	0.3362	0.6716 to 3.250	1.182	0.708	0.5018 to 2.789	1.179	0.5575	0.6773 to 2.076
**S100A10 methylation RFS TCGA**									
	high vs. low			high vs. intermediate			low vs. intermediate		
Probe	HR	p-value	C.I.	HR	p-value	C.I.	HR	p-value	C.I.
cg13249591	2.441	0.011	1.209 to 3.927	2	0.054	1.004 to 3.561	1.372	0.1774	0.8667 to 2.189
cg13445177	2.375	0.0155	1.154 to 3.486	2.25	0.0317	1.082 to 3.977	0.9959	0.9849	0.6469 to 1.533
cg18348690	1.768	0.1156	0.8873 to 3.070	1.494	0.2827	0.7393 to 2.827	1.164	0.5211	0.7330 to 1.848
cg26230275	2.009	0.0636	0.9748 to 3.452	1.835	0.1133	0.8850 to 3.281	0.9221	0.7231	0.5796 to 1.455
cg20994097	2.053	0.1329	0.8360 to 4.379	1.683	0.2819	0.6841 to 3.954	1.256	0.4758	0.6690 to 2.392
cg06698332	3.723	0.0187	1.206 to 6.208	1.787	0.3323	0.6048 to 4.713	1.68	0.0936	0.9248 to 3.218
**S100A10 methylation OS ICGC**									
	high vs. low			high vs. intermediate			low vs. intermediate		
Probe	HR	p-value	C.I.	HR	p-value	C.I.	HR	p-value	C.I.
cg13249591	2.63	< 0.0001	1.510 to 3.331	1.862	0.0134	1.133 to 2.699	1.521	0.0052	1.144 to 2.097
cg13445177	2.666	0.0006	1.393 to 3.298	2.372	0.0025	1.282 to 3.134	1.167	0.302	0.8706 to 1.573

Supplemental Table 10. Multiple comparisons of OS and RFS using the mRNA Ternary classifier. Multiple comparisons of S100A10 methylation survival functions were performed on the TCGA and ICGC patient cohorts. *P*-values were adjusted to the Bonferroni-corrected threshold. Adjusted *p*-value is *p*-value/K = 0.017 where K=3 and represents the number of comparisons made.

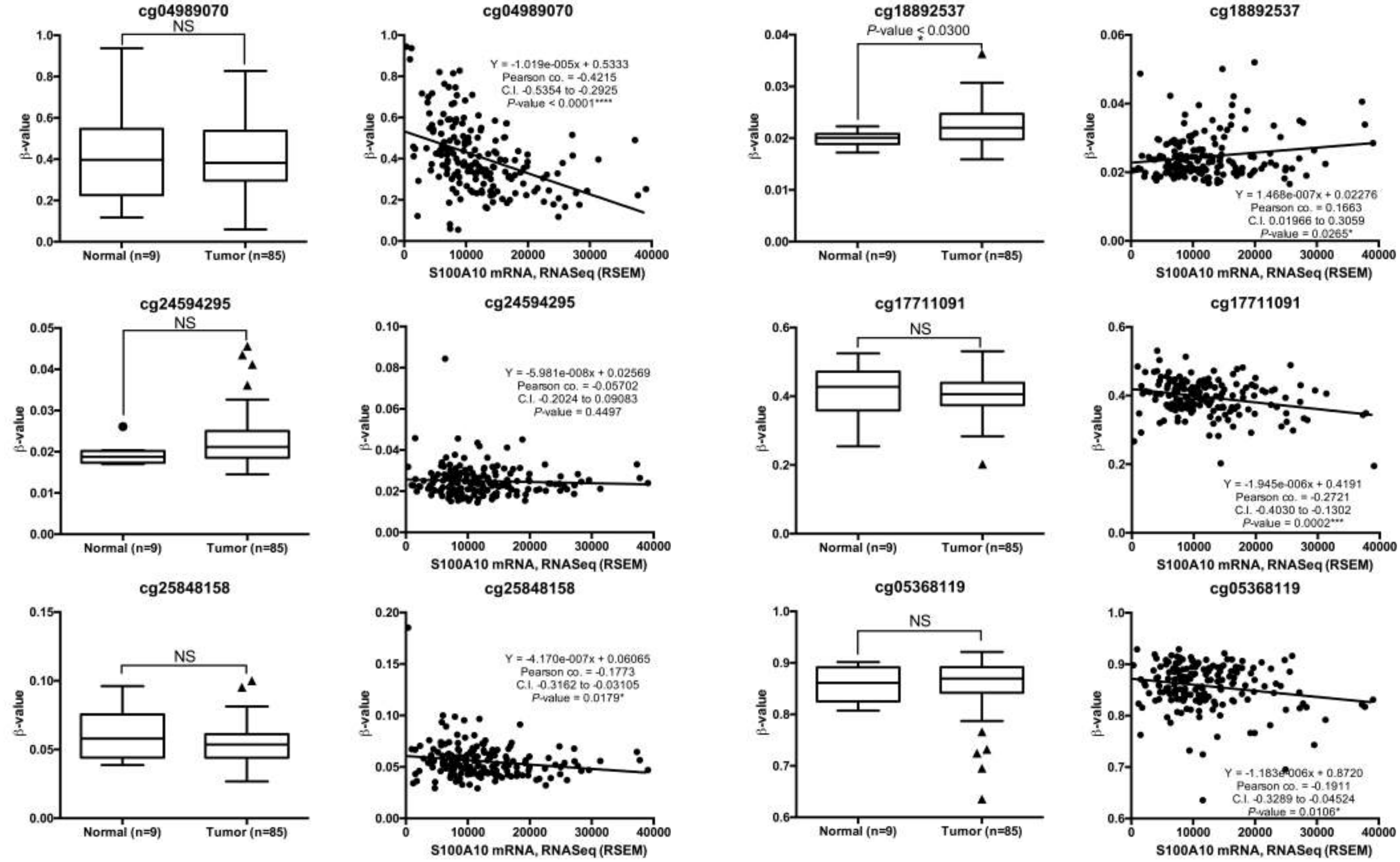

Supplemental Figure 6. The β values of probes that were not differentially-methylated and/or did not negatively correlate with *S100A10* mRNA expression. For normal vs. tumor comparisons, the raw data was extracted from MethHC (http://methhc.mbc.nctu.edu.tw/php/index.php), described by Huang *et al*. (2015). Nucleic Acids Res. (database issue):D856-61. The β values of each probe were assessed in 85 PDAC tumors and 9 normal tissues (first and third columns). For mRNA vs. methylation correlations, raw β values of individual probes were extracted from Maplab Wanderer (http://maplab.imppc.org/wanderer/) (Villanueva *et al*. 2015); Epigenetics Chromatin. 8:22 (eCollection 2015) and plotted against RNA Seq V2 (RSEM) expression values of S100A10 in matched patients. Pearson correlation was used to generate correlation graphs of β values and S100A10 mRNA expression (second and fourth columns). Tukey comparison was used to plot raw β values in normal and tumor tissues (*p*-value: 0.05).

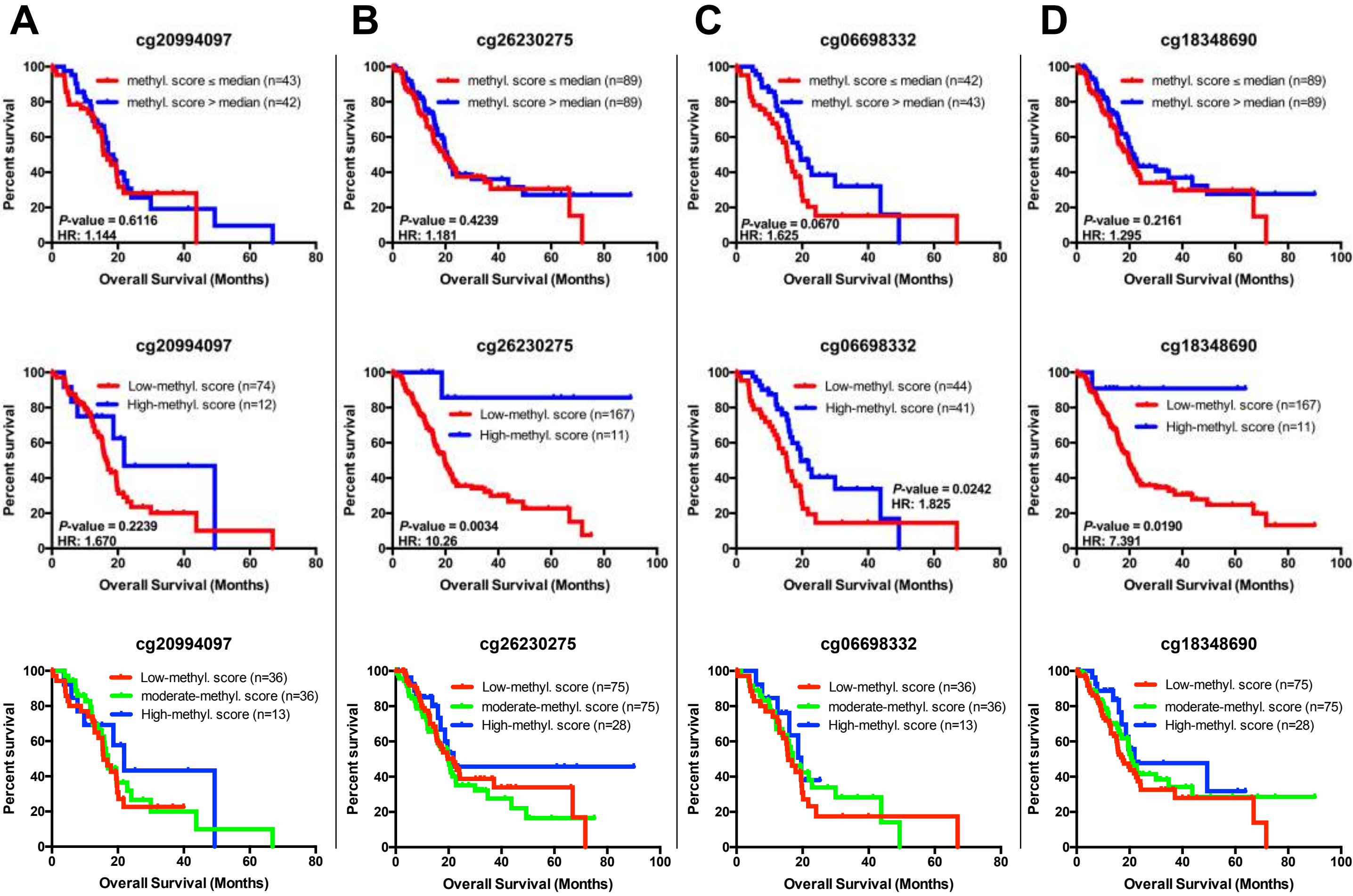

Supplemental Figure 7. Kaplan Meier survival analyses of OS based on β values of the remaining four probes in the TCGA PDAC cohort. Kaplan Meier (KM) plots of overall survival (n=178) based on β values of the (A) cg20994097, (B) cg26230275, (C) cg06698332 and (D) cg18348690. The same three-tier method of classification was used; A median cut-off (top), best cut-off (middle), and a ternary cut-off (bottom). Raw β values of individual probes were extracted from Maplab Wanderer (Villanueva *et al*. 2015). Epigenetics Chromatin. 8:22 (eCollection 2015) matched with OS of TCGA PDAC patients. β values for probes cg20994097 and cg06698332 were available for 85 patients only.

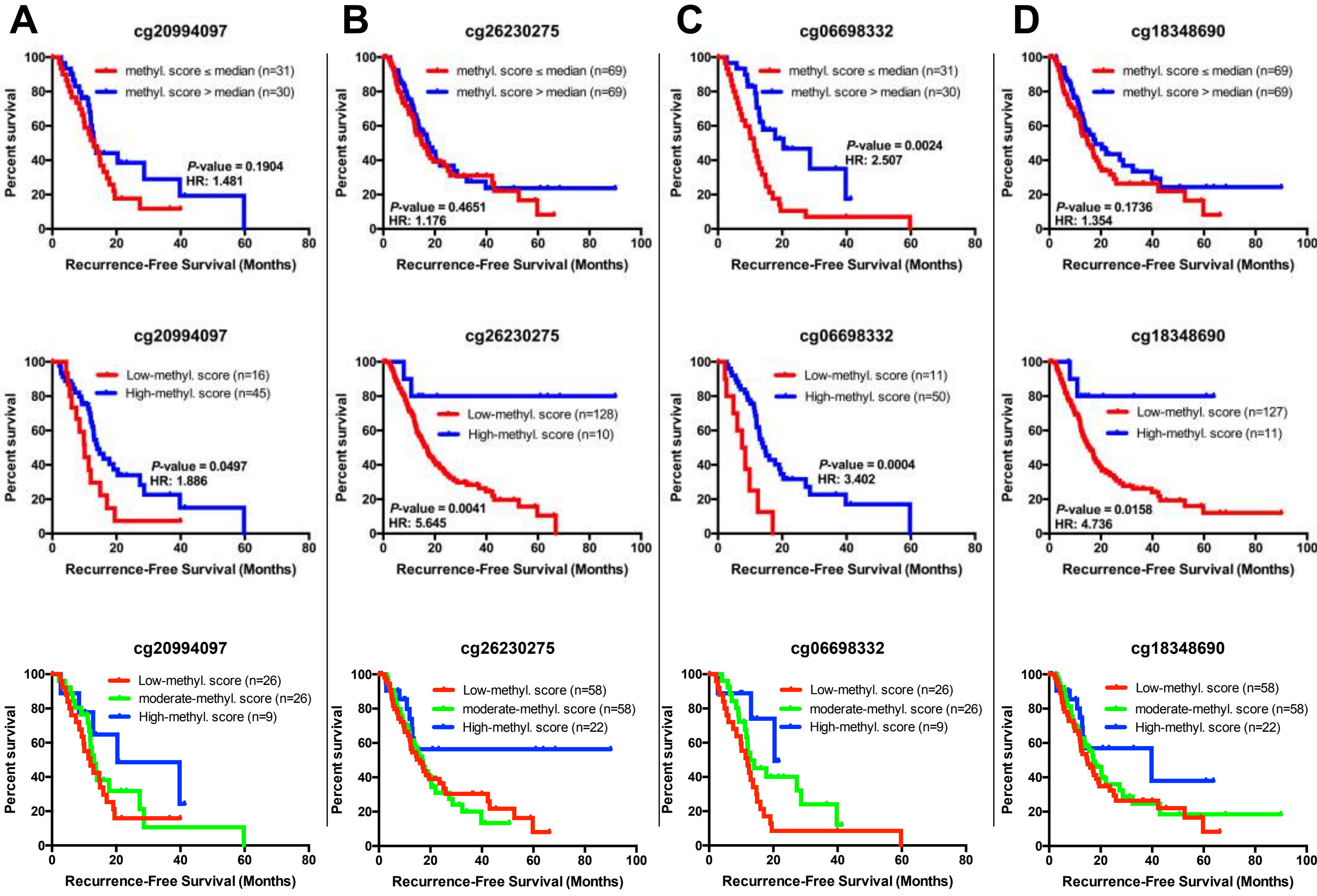

Supplemental Figure 8. Kaplan Meier survival analyses of RFS based on β values of the remaining four probes in the TCGA PDAC cohort. Kaplan Meier (KM) plots of recurrence-free survival (n=138) based on β values of the (A) cg20994097, (B) cg26230275, (C) cg06698332 and (D) cg18348690. The same three-tier method of classification was used; A median cut-off (top), best cut-off (middle), and a ternary cut-off (bottom). Raw β values of individual probes were extracted from Maplab Wanderer (Villanueva *et al*. 2015). Epigenetics Chromatin. 8:22 (eCollection 2015) matched with RFS of TCGA PDAC patients. β values for probes cg20994097 and cg06698332 were available for 61 patients only.

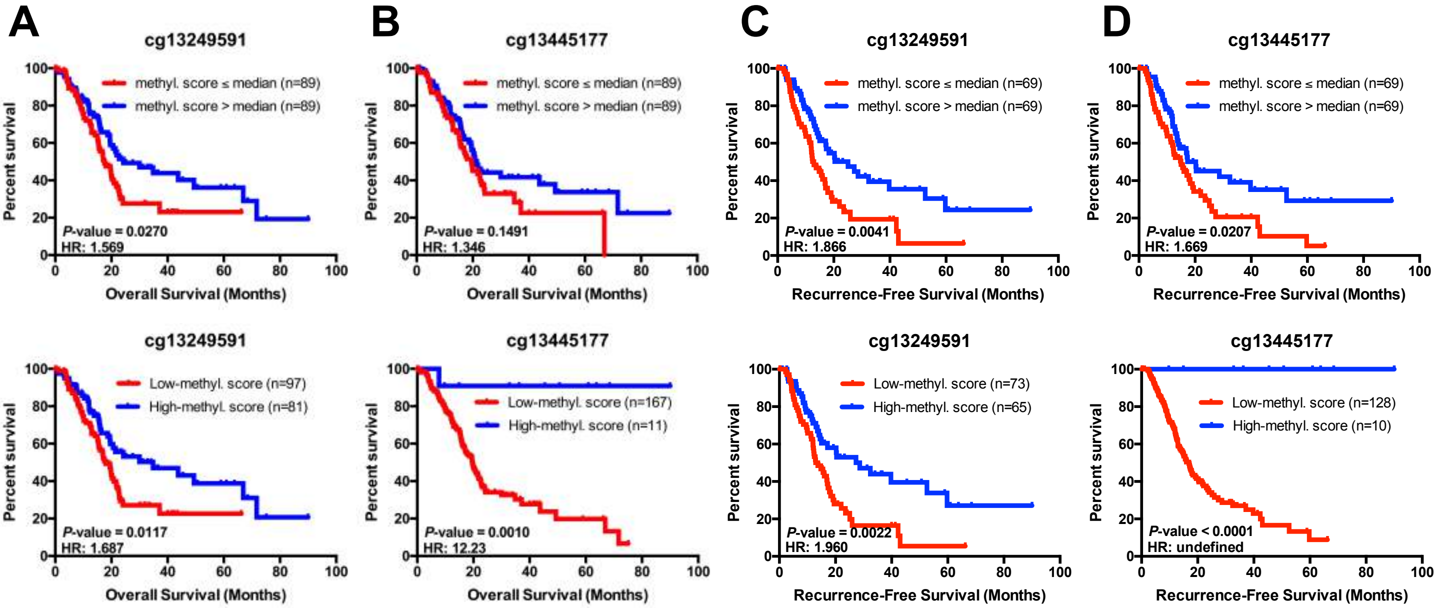

Supplemental Figure 9. Kaplan Meier analyses of CpG islands corresponding to probes cg13249591 and cg13445177 using median and optimal cut-offs. Kaplan Meier (KM) plots of (A, B) overall survival (n=178) and (C, D) recurrence-free survival (n=139) based on β values of the (A, C) cg13249591 and (B, D) cg13445177 CpG sites.

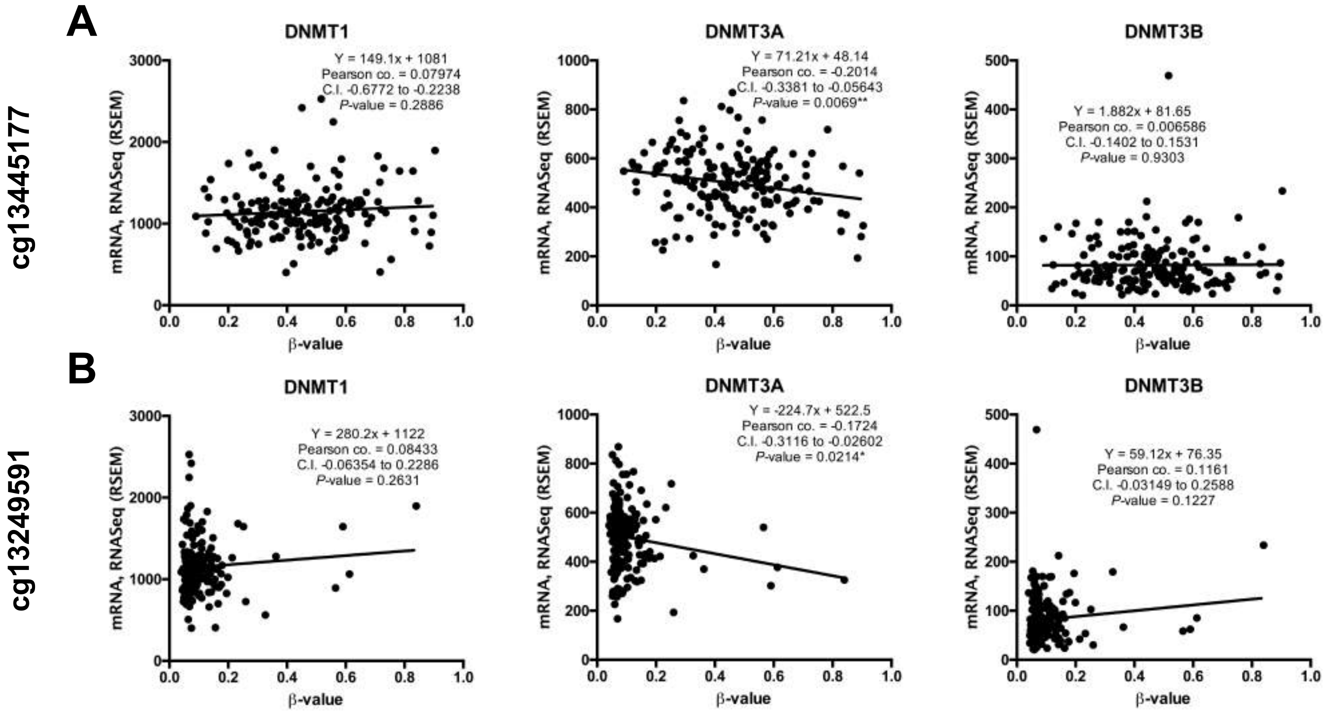

Supplemental Figure 10. The β values of the probes cg13445177 and cg13249591 do not positively correlate with mRNA expression of *de novo* methyltransferases. Raw β values of cg13445177 and cg13249591 were extracted from Maplab and plotted against RNA Seq V2 (RSEM) expression values of the *de novo* methyltransferases *DNMT1*, *DNMT3B* and *DNMT3A* (from Cbioportal).

A

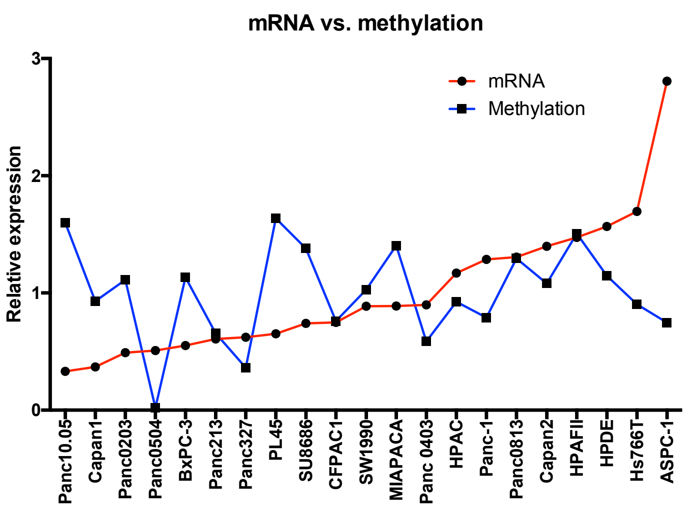

B

Analyzed Sequence (377 nt)

TC[1]**CG**CC[2]**CG**CCT[3]**CG**GCCTCCCAAAGTGTTGGGATTACAGG[4]**CG**TGAGCCAC[5]**CG**[6]**CG**CC[7]**CG**GCCAGTTTTTAACAC
TATTAGCCACACTGAAACTGAACTATTGATCAAGTGA[8]**CG**CCACACAAAGGGGTAAATCCCCTGTTCAACAAAGGG
TTTGTGA[9]**CG**CCCCTGGGTGCTGACAAGCCAAAC[10]**CG**CACCCTCCCTG[11]**CG**GCACCT[12]**CG**[13]**CG**GGC[14]**CG**GTGGGG[15]
CGGGAAGCC[16]**CG**GCTTCTGGGGAGGTGC[17]**CG**CCCCTCCACTGG[18]**CG**CAGGC[19]**CG**C[20]**CG**AGACCCCCAGA[21]**CG**GAC
CTCCTAGGGCTAATCTGATAGTGCCTCTGAGGT[22]**CG**ATAGGACTCCA[23]**CG**TGCCACTCCCTGCAGGGTCATCCAGCA
AGTAATTCCTAGACC[24]**CG**

HM450 Array Probe: cg20167074; cg13445177; cg13249591; cg25848158; cg24594295

C

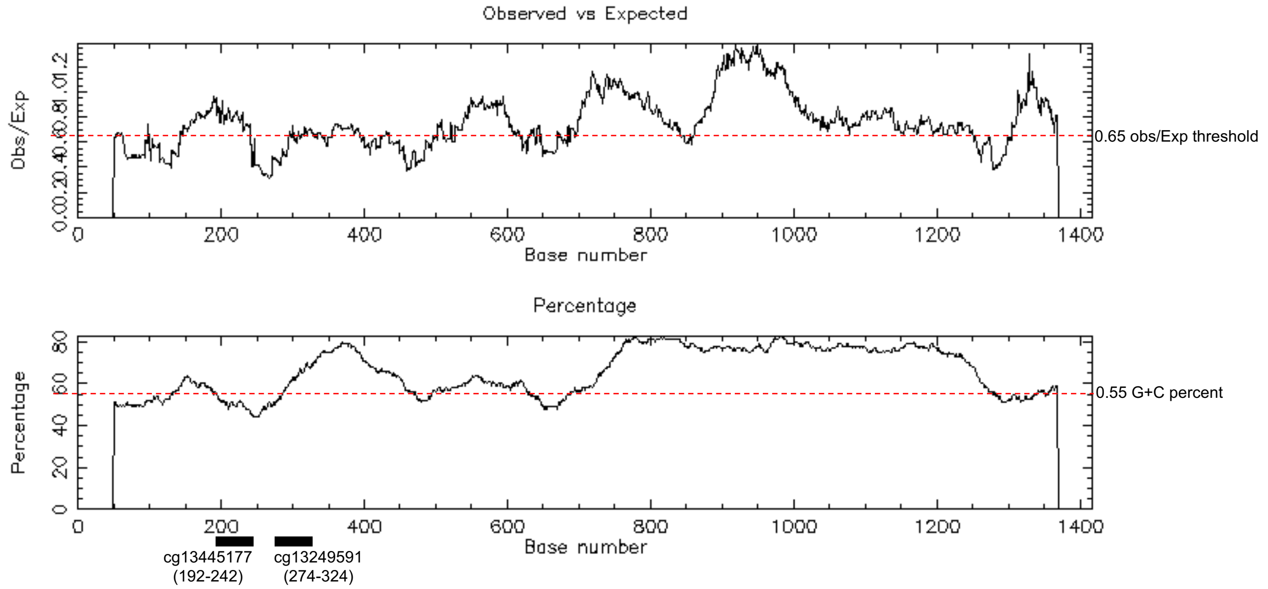

Supplemental Figure 11. S100A10 promoter methylation. (A) Analysis of relative mRNA and methylation scores of the 21 PDAC cell lines in CCLE. The CCLE expression values were normalized to the average of the mRNA and methylation scores respectively to allow single-axis plot. (B) The 377-nucleotide promoter region of S100A10 used for pyrosequencing. The sequence highlights the sequenced CpG sites as well the location of HM450 methylation probes (as highlighted). The beginning of exon1 is underlined. (C) Promoter CpG island analysis using EMBOSS CpGplot tool from the EMBL-EBI database: (https://www.ebi.ac.uk/Tools/seqstats/emboss_cpgplot/). The CpG island criteria set by Takai and Jones (2002) were used. These include: 1) minimum length of an island is 500bp. 2) Minimum observed/expected is the minimum average observed to expected ratio of C plus G to CpG in a set of 10 windows that are required before a CpG island is reported. The threshold value is 0.65. 3) Minimum percentage is minimum average percentage of G plus C a set of 10 windows that are required before a CpG island is reported. The threshold value is 0.55.

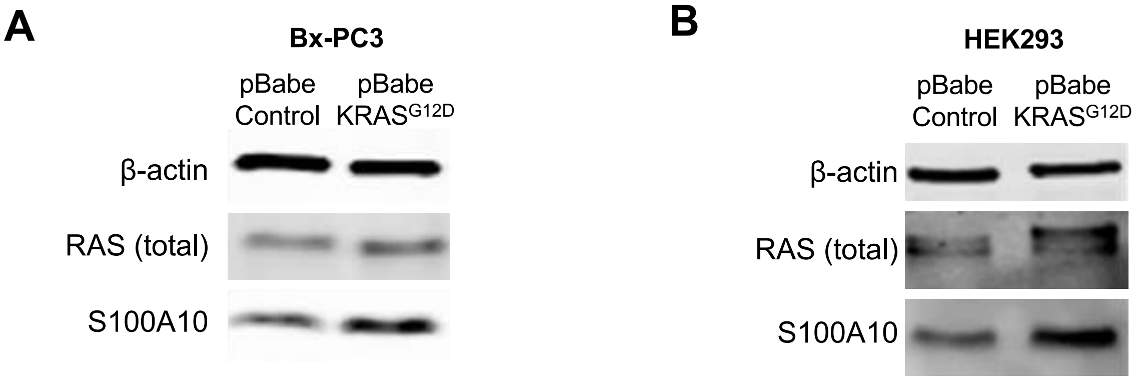

Supplemental Figure 12. Effect of oncogenic KRAS^{G12D} on S100A10 expression in WT-KRAS cells. Western blot analysis of S100A10 protein in BxPC-3 (A) and HEK293 (B) cells which were stably transfected with the pBabe Control and pBabe KRAS^{G12D} vectors.

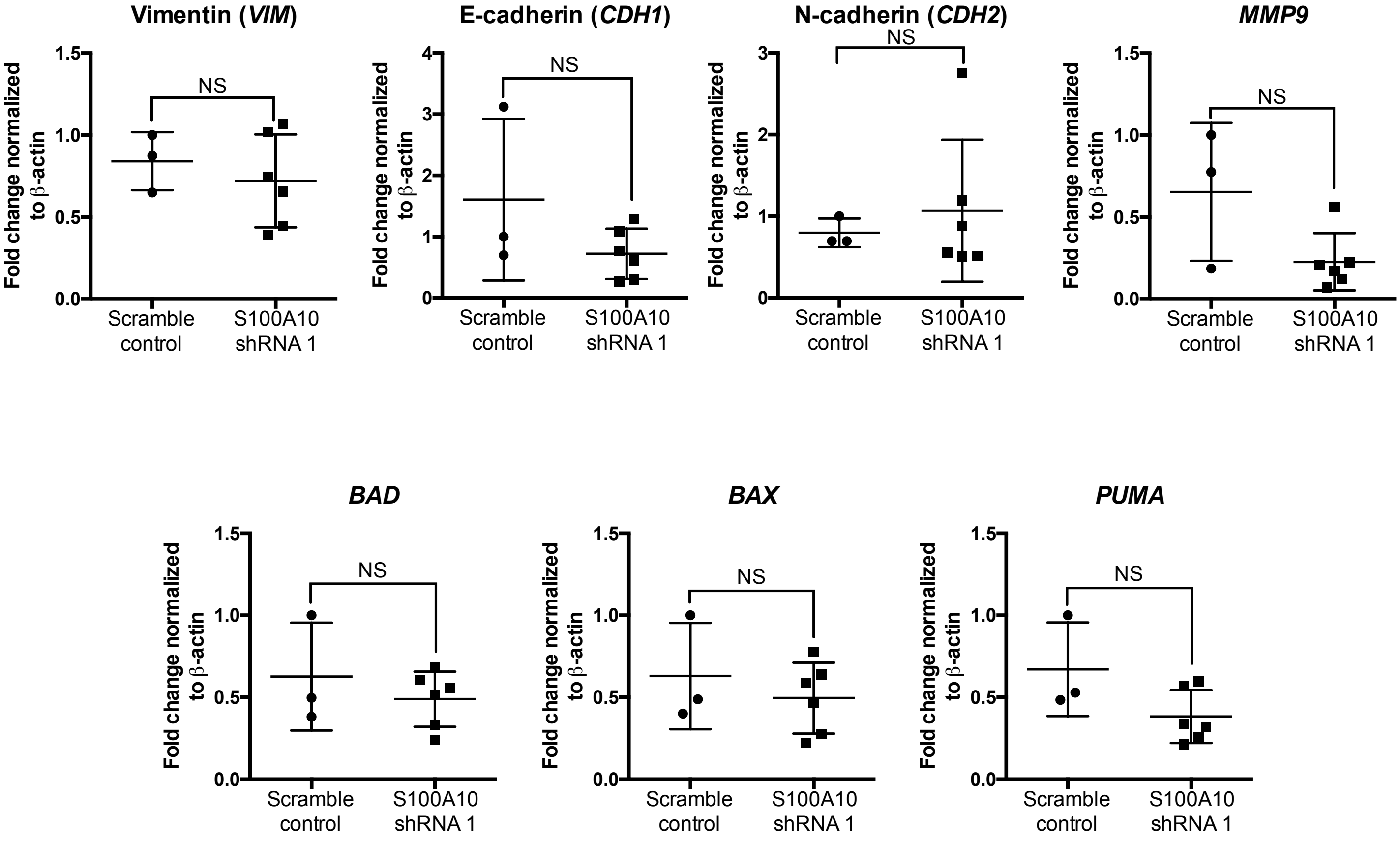

Supplemental Figure 13. RT-qPCR of several genes in scramble control and S100A10-shRNA 1 Panc-1 tumors. These genes were not significantly altered by S100A10 depletion in Panc-1 tumors.

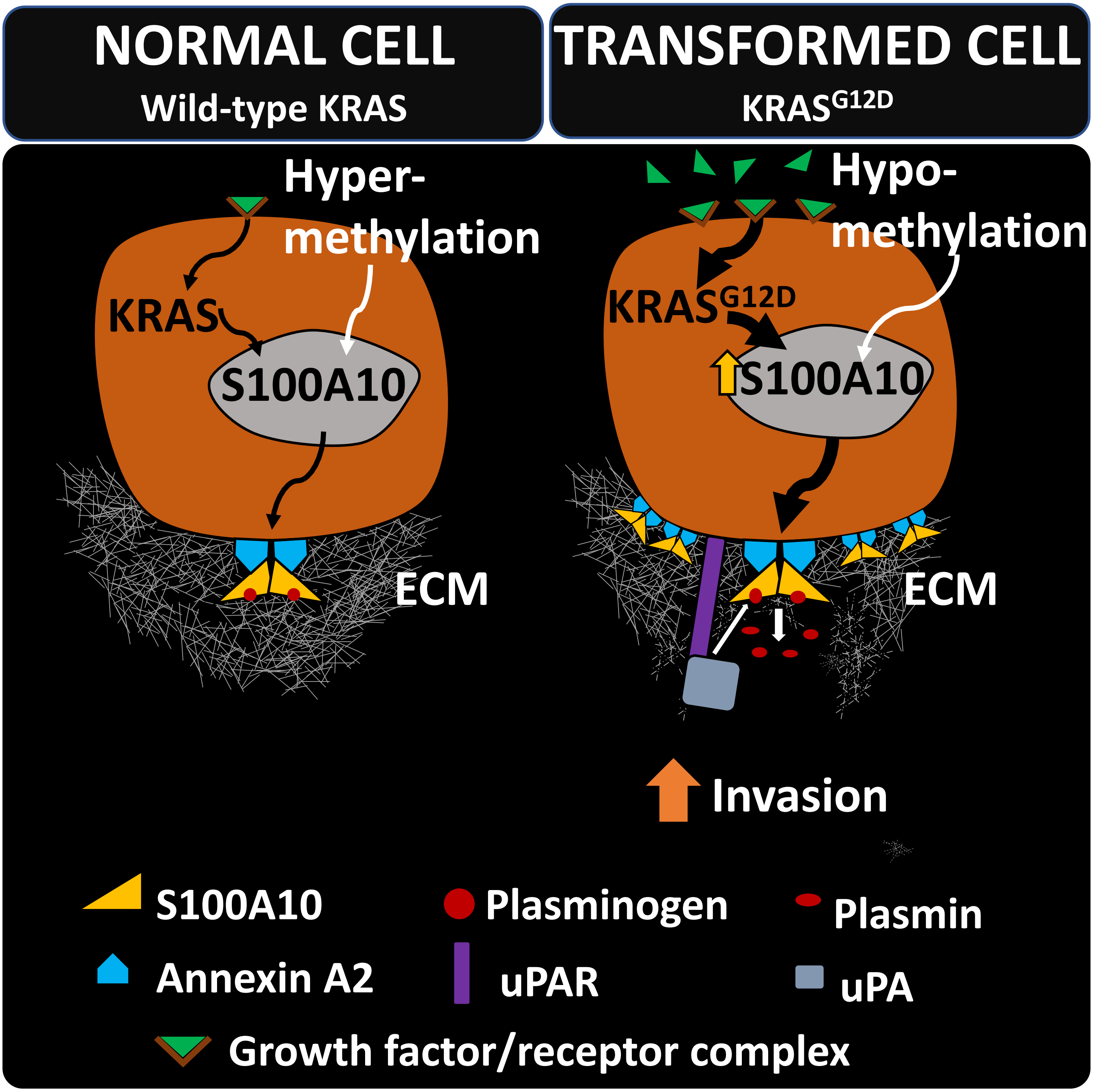

Supplemental Figure 14. Schematic representation of $KRAS^{G12D}$- and methylation-mediated regulation of S100A10-dependent plasminogen activation. Oncogenic KRAS induces S100A10 upregulation which in turn contributes to plasminogen activation and plasminogen-dependent invasion. The expression of S100A10 was also driven by DNA methylation of its promoter region. A heterotetrameric complex is formed of two annexin A2 subunits and 2 subunits of S100A10 (dimer). KRAS is also capable of upregulating uPA and uPAR whose localization is induced by S100A10 binding to plasminogen. The latter is activated into plasmin which cleaves extracellular matrix (ECM) proteins and destabilizes its structure allowing pancreatic cancer cell advancement.

Gene	Primer	Sequence
ACTB (β-Actin)	Forward	CTTCCAGCCTTCCTTCCTGG
	Reverse	CTGTGTTGGCGTACAGGTCCT
S100A10	Forward	CCCTCTGGCTGTGGACAAAA
	Reverse	CGACCCTTTGGGACAACTCT
VEGFA	Forward	CTTGCCTTGCTGCTCTACCT
	Reverse	GCAGTAGCTGCGCTGTGATAGA
CCND1 (Cyclin D1)	Forward	GATGCCAACCTCCTCAACGA
	Reverse	GGAAGCGGTCCAGGTAGTTC
VIM (Vimentin)	Forward	TCTACGAGGAGGAGATGCGG
	Reverse	GGTCAAGACGTGCCAGAGAC
CDH1 (E-Cadherin)	Forward	TCATGAGTGTCCCCCGGTAT
	Reverse	TCTTGAAGCGATTGCCCCAT
CDH2 (N-Cadherin)	Forward	AGCCTGACACTGTGGAGCCT
	Reverse	TCAGCGTGGATGGGTCTTTC
MMP9	Forward	CAAGGACCGGTTTATTTGGC
	Reverse	ATTCCCTGCGAAGAACACAGC
BAD	Forward	GGTTCTGAGGGGAGACTGAGGT
	Reverse	ACTCGGCTCAAACTCTGGGA
BAX	Forward	CAGGGGCCCTTTTGCTTCAG
	Reverse	TAGAAAAGGGCGACAACCCG
PUMA	Forward	CTCCTCTCGGTGCTCCTTCA
	Reverse	CTCTCTCTAAACCTATGCAATGGGA
**Primers for bisulfite conversion and pyrosequencing**		
S100A10	Forward	TGGTTAAGTTGGTGTTGAATTT
	Reverse[b]	AATAACCCTACAAAAAATAACA[b]
	Forward[Seq]	AATTTTTGATTTGAGGTGA
p-SUPER shRNA		
S100A10	dsDNA oligo	5’-GAT CCC CGT GGG CTT CCA GAG CTT CTT TCA AGA GAA GAA GCT CTG GAA GCC CAC TTT TTA-3’
		5’-AGC TTA AAA AGT GGG CTT CCA GAG CTT CTT CTC TTG AAA GAA GCT CTG GAA GCC CAC GGG-3’

Supplemental Table 11. List of primer sequences used in RT-qPCR and pyrosequencing as well as dsDNA oligo used for S100A10 shRNA. [b] represents biotinylated primers. ”Seq” is used for the pyrosequencing step along with the biotinylated reverse primer.
